# Phenylpropionamides, Piperidine, and Phenolic Derivatives from the Fruit of *Ailanthus altissima*

**DOI:** 10.3390/molecules22122107

**Published:** 2017-12-04

**Authors:** Jian-Cheng Ni, Jian-Ting Shi, Qing-Wei Tan, Qi-Jian Chen

**Affiliations:** 1 Key Laboratory of Bio-Pesticide and Chemistry-Biology, Ministry of Education, Fujian Agriculture and Forestry University, Fuzhou 350002, China; njc2130215001@163.com (J.-C.N.); shijianting00@163.com (J.-T.S.); 2 Key Laboratory of Plant Virology of Fujian Province, Institute of Plant Virology, Fujian Agriculture and Forestry University, Fuzhou 350002, China

**Keywords:** *Ailanthus altissima*, Simaroubaceae, phenylpropionamide, piperidine, phenols, flavonoid, *Tobacco mosaic virus* (TMV)

## Abstract

Four novel compounds—two phenylpropionamides, one piperidine, and one phenolic derivatives—were isolated and identified from the fruit of a medicinal plant, *Ailanthus altissima* (Mill.) Swingle (Simaroubaceae), together with one known phenylpropionamide, 13 known phenols, and 10 flavonoids. The structures of the new compounds were elucidated as 2-hydroxy-*N*-[(2-*O*-β-d-glucopyranosyl)phenyl]propionamide (**1**), 2-hydroxy-*N*-[(2-*O*-β-d-glucopyranosyl-(1→6)-β-d-glucopyranosyl)phenyl]propionamide (**2**), 2β-carboxyl-piperidine-4β-acetic acid methyl ester (**4**), and 4-hydroxyphenyl-1-*O*-[6-(hydrogen-3-hydroxy-3-methylpentanedioate)]-β-d-glucopyranoside (**5**) based on spectroscopic analysis. All the isolated compounds were evaluated for their inhibitory activity against *Tobacco mosaic virus* (TMV) using the leaf-disc method. Among the compounds isolated, arbutin (**6**), β-d-glucopyranosyl-(1→6)-arbutin (**7**), 4-methoxyphenylacetic acid (**10**), and corilagin (**18**) showed moderate inhibition against TMV with IC_50_ values of 0.49, 0.51, 0.27, and 0.45 mM, respectively.

## 1. Introduction

*Ailanthus altissima* (Mill.) Swingle (Simaroubaceae), a deciduous tree (6–20 m in height), is native to Mainland China and now naturalized in many temperate regions of the world [1,2]. The stem and root bark have been used as traditional Chinese medicines for the treatment of colds, bleeding, and gastric diseases [3,4]. Phytochemical studies, especially on the stem and root bark of *A. altissima*, have led to the characterization of quassinoids [5,6], alkaloids [7,8], triterpenoids [9,10], coumarins [9], lignans [11], as well as sterols, lipids, and other phenolic derivatives [12]. However, little is known concerning the constituents of the fruit of *A. altissima*, which was also used as traditional Chinese medicine for bleeding and antibacterial. By far, previous phytochemical studies have demonstrated the identification of only four quassinoid glycosides [13], and several stigmasterols [14,15] from the fruit. We report in this paper the isolation and structure elucidation of four novel compounds—two phenylpropionamides (**1** and **2**), one piperidine (**4**) and one phenolic (**5**) derivatives—as well as 24 known constituents—one known phenylpropionamide (**3**), 13 phenols (**6**–**18**), and 10 flavonoids (**19**–**28**). All compounds were investigated for their anti-*Tobacco mosaic virus* (TMV) activity.

## 2. Results

### 2.1. Extraction, Isolation, and Sructure Elucidation

**Figure 1:**
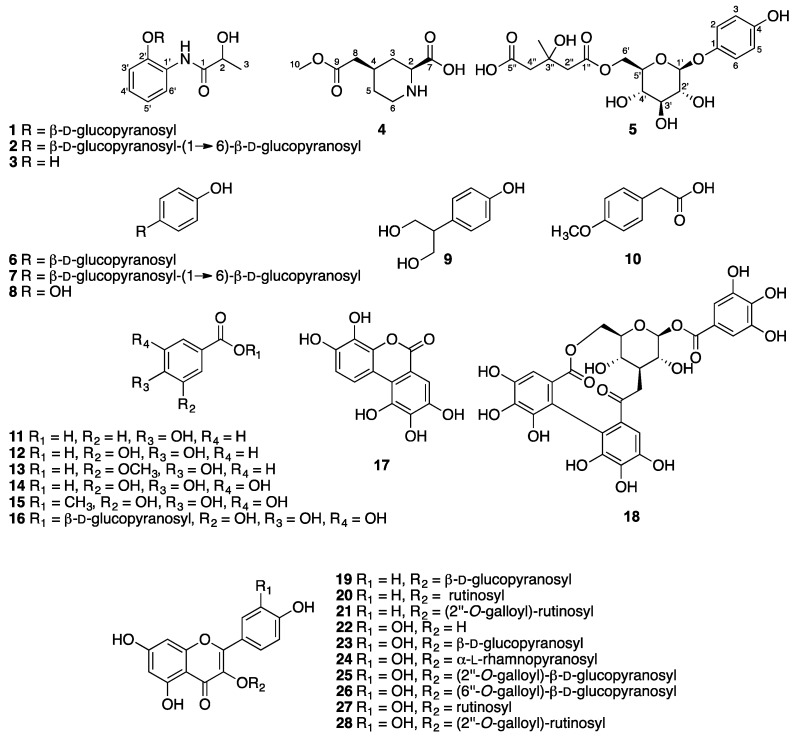
Chemical structures of **1**–**28**.

Chromatographic purification of the *n*-BuOH-soluble fraction from MeOH extract of the dried *A. altissima* fruit afforded 28 compounds, including four new ones (**1**, **2**, **4**, and **5**, Figure 1).

**Figure 2:**
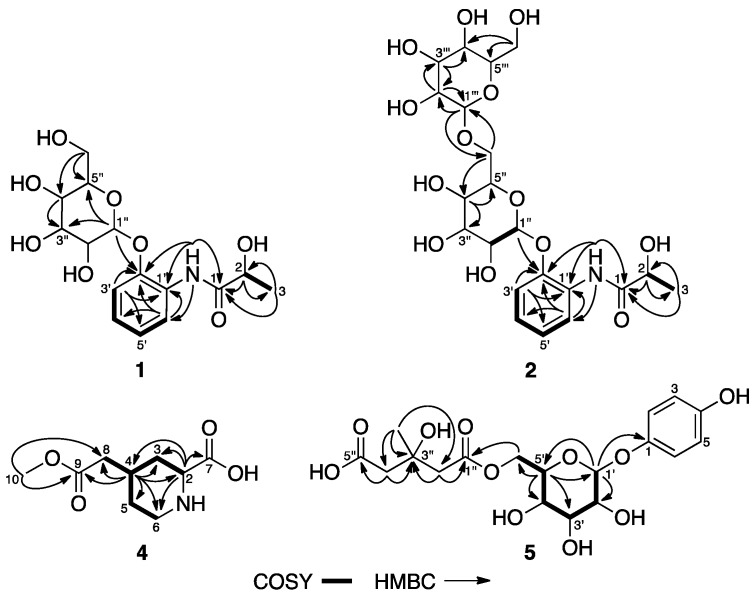
Selected ^1^H-^1^H COSY and HMBC correlations of **1**, **2**, **4**, and **5**.

Compound **1** was isolated as a white amorphous powder. It was assigned with a molecular formula of C_15_H_21_NO_8_ by an HR-ESI-MS (high resolution electrospray ionization mass spectrometry) ion peak at *m*/*z* = 366.1185 [M + Na]^+^ (Calcd. for C_15_H_21_NO_8_Na, 366.1159). The IR spectrum (Supplementary Materials) exhibited absorption bands due to the presence of hydroxyl, amide, and phenyl groups (3478, 3388, 1674, 1603, 1534, and 1456 cm^−1^). The ^1^H-NMR (Table 1) and HSQC (heteronuclear single quantum coherence) spectrum of **1** indicated the presence of a 1,2-disubstituted benzene ring [*δ*_H_ 8.23 (1H, dd, *J* = 7.4, 2.3 Hz), 7.20 (1H, dd, *J* = 7.5, 2.0 Hz), 7.05 (1H, td, *J* = 7.5, 2.0 Hz), and 7.02 (1H, td, *J* = 7.5, 1.7 Hz)], an oxygenated methine [4.15 (1H, qd, *J* = 6.8, 5.1 Hz)], a methyl [*δ*_H_ 1.33 (3H, d, *J* = 6.8 Hz)], an amide proton [*δ*_H_ 9.42 (1H, s)], and a glucopyranosyl moiety [*δ*_H_ 4.85 (1H, d, *J* = 7.5 Hz), 3.69 (1H, ddd, *J* = 11.8, 5.4, 2.2 Hz), 3.49 (1H, dt, *J* = 11.8, 5.9 Hz), 3.27–3.32 (3H, overlap), and 3.18 (1H, td, *J* = 9.2, 5.4 Hz)]. Its ^13^C-NMR (Table 1) and DEPT (distortionless enhancement by polarization transfer) spectra showed 15 carbon resonances, including one methyl, one methylene, 10 methines, and three quaternary carbons (including one carbonyl). The HMBC (heteronuclear multiple bond correlation) correlations (Figure 2) observed from the amide proton to C-1 (*δ*_C_ 173.1), C-1′ (*δ*_C_ 128.6), C-2′ (*δ*_C_ 146.5), and C-6′ (*δ*_C_ 119.6) and from H-2 to C-1, C-2 (*δ*_C_ 67.8), and C-3 (*δ*_C_ 20.8) indicated the presence of a 2-hydroxypropionamide moiety, which was attached to C-1′ of the benzene ring via an NH linkage. The anomeric proton appearing as a doublet at *δ*_H_ 4.85 with a diaxial coupling constant of 7.5 Hz suggested that the glucopyranosyl moiety must be a β-anomer. Additionally, it was attached to C-2′ of the benzene ring through an oxygen, as indicated from the HMBC correlations from the anomeric proton to C-2′, which was further confirmed by the NOESY (nuclear Overhauser effect spectroscopy) correlation between H-3′/H-1‴. The acid hydrolysis of **1** afforded d-glucose, which was identified using TLC by comparison with standard sugars. Therefore, the structure of Compound **1** was established as 2-hydroxy-*N*-[(2-*O*-β-d-glucopyranosyl)phenyl]propionamide.

Compound **2** was obtained as a white amorphous powder. Its molecular formula was deduced to be C_21_H_31_NO_13_ by an HR-ESI-MS ion peak at *m*/*z* 528.1726 [M + Na]^+^ (Calcd. for C_21_H_31_NO_13_Na, 528.1688). The IR spectrum (Supplementary Materials) exhibited absorption bands due to the presence of hydroxyl, amide, and phenyl groups (3423, 3346, 1662, 1602, 1532, and 1454 cm^−1^). Analysis of the ^1^H- and ^13^C-NMR data (Table 1) suggested that Compound **2** possessed the same aglycone as that of **1**, which was confirmed by the observed HMBC correlations as shown in Figure 2. The ^1^H-NMR spectra of **2** showed two anomeric proton signals at *δ*_H_ 4.86 (1H, d, *J* = 7.4 Hz, H-1″) and 4.22 (1H, d, *J* = 7.8 Hz, H-1‴), which correlated to the corresponding anomeric carbon signals at *δ*_C_ 101.5 (C-1″) and 103.2 (C-1‴) in the ^13^C-NMR spectra, respectively, indicating the presence of two glucopyranosyl units with β-form. The HMBC correlations from an anomeric proton at *δ*_H_ 4.22 (H-1‴) to *δ*_C_ 68.3 (C-6″) and from another anomeric proton at *δ*_H_ 4.86 (H-1″) to *δ*_C_ 146.4 (C-2′) indicated that two sugars were connected through 1→6 linkage, and the sugar chain was attached at C-2′ of the aglycone through an oxygen. Therefore, Compound **2** was determined as 2-hydroxy-*N*-[(2-*O*-β-d-glucopyranosyl-(1→6)-β-d-glucopyranosyl)phenyl]propionamide.

Compound **3** was obtained as a white amorphous powder, with a molecular formula of C_9_H_12_NO_3_ as indicated by an ion peak at *m*/*z* 182.0840 [M + Na]^+^ (Calcd. for C_9_H_12_NO_3_Na, 182.0812) in its HR-ESI-MS. Comparison of the ^1^H- and ^13^C-NMR data with that of Compounds **1** and **2**, as well as with those reported in the literature [16], indicated that Compound **3** was the aglycone of Compounds **1** and **2** with a known structure, 2-hydroxy-*N*-(2-hydroxyphenyl)propionamide.

**Figure 3:**
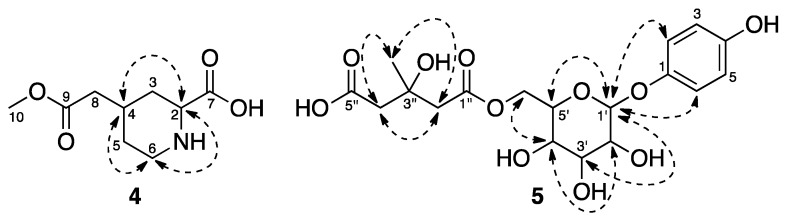
Key NOESY correlations of **4** and **5**.

Compound **4** was isolated as a white amorphous powder. It was assigned with a molecular formula of C_9_H_15_NO_4_ by an HR-ESI-MS ion peak at *m*/*z* = 224.0907 [M + Na]^+^ (Calcd. for C_9_H_15_NO_4_Na, 224.0899). Its IR spectrum (Supplementary Materials) showed the presence of a secondary amide (1624 cm^−1^), a carbonyl (1729 cm^−1^), and a strong peak for a hydroxyl group (3428 cm^−1^). The ^1^H-NMR (Table 1) and HSQC spectrum of **4** revealed the presence of one methoxyl at *δ*_H_ 3.66 (3H, s), two methines at *δ*_H_ 3.47 (1H, dd, *J* = 12.8, 3.2 Hz) and 2.11 (1H, m), as well as four methylene protons. The ^13^C-NMR spectrum and DEPT showed the presence of two methine carbons at *δ*_C_ 60.6 and 32.9, one methoxyl carbon at *δ*_C_ 52.1, two carbonyl carbons at *δ*_C_ 173.7 and 173.8, as well as four methylene carbons. The key HMBC correlations (Figure 2) from H-2 to C-3, C-4, and C-6 and from H-4 to C-2, C-3, C-5, and C-6 revealed the presence of a 2,4-disubstituted piperidine ring, which was confirmed by ^1^H-^1^H COSY (correlated spectroscopy) correlations between H-2/H-3, H-3/H-4, H-4/H-5, H-5/H-6, and H-4/H-8. An acetic acid methyl ester group was established by the HMBC correlations from the methoxyl protons to C-8 and C-9, and it was attached to C-4 of the piperidine ring as indicated by the HMBC correlations from H-4 to C-8 and C-9. The HMBC correlation from H-2 to C-7 proved that a carbonyl group was located at C-2. The NOESY correlations (Figure 3) between H-2/H-4, H-2/H-6, and H-4/H-6 indicated that the two substituents were *cis* to each other. Thus, Compound **4** was determined as 2β-carboxyl-piperidine-4β-acetic acid methyl ester.

Compound **5** was obtained as a pale-yellow solid. Its molecular formula C_18_H_24_O_11_ was established by HR-ESI-MS at *m*/*z* = 439.1234 [M + Na]^+^ (Calcd. for C_18_H_24_O_11_Na, 439.1211). The IR spectrum (Supplementary Materials) of **5** showed strong absorption bands at 3420 and 1718 cm^−1^, suggesting the presence of hydroxyl and carbonyl groups, respectively. Its ^1^H-NMR and HSQC spectrum (Supplementary Materials) showed signals indicating the presence of a 1,4-disubstituted benzene ring [*δ*_H_ 6.84 (2H, d, *J* = 9.0 Hz, H-2 and H-6) and 6.66 (2H, d, *J* = 9.0 Hz, H-3 and H-5)], a glucopyranosyl moiety [4.65 (1H, d, *J* = 7.7 Hz, H-1′), 4.32 (1H, dd, *J* = 11.8, 2.0 Hz, H-6′), 4.00 (1H, dd, *J* = 11.8, 7.0 Hz, H-6′), 3.50 (1H, ddd, *J* = 9.2, 7.0, 2.0 Hz, H-5′), 3.26 (1H, t, *J* = 8.8 Hz, H-3′), 3.19 (1H, dd, *J* = 8.8, 7.7 Hz, H-2′), and 3.14 (1H, t, *J* = 9.2 Hz, H-4′)], one methyl [1.22 (3H, s, H-6″)], and two methylene groups [2.57 (1H, d, *J* = 14.0 Hz, H-2″), 2.49 (1H, overlap, H-2″), 2.40 (1H, d, *J* = 15.0 Hz, H-4″), and 2.33 (1H, d, *J* = 15.0 Hz, H-4″)]. The ^13^C-NMR and DEPT of **5** displayed resonances of two carbonyls [*δ*_C_ 174.4 (C-5″) and 170.5 (C-1″)], one oxygenated quaternary carbon [*δ*_C_ 68.9 (C-3″)], two methylenes [*δ*_C_ 46.1 (C-4″) and 45.9 (C-2″)], and one methyl [*δ*_C_ 27.6 (C-6″)] carbons, besides signals for a typical glucopyranosyl moiety and a *p*-hydroxyphenyl group. The HMBC correlations (Figure 2) observed from H_2_-2″ to C-1″ and C-3″ and from H_2_-4″ to C-3″ and C-5″, as well as from H_3_-CH_3_ to C-2″, C-3″, and C-4″, allowed for the establishment of a 3-hydroxy-3-methyl glutaryl (HMG) group. The HMBC correlations from the anomeric proton H-1′ to C-1 (*δ*_C_ 150.1) and from H_2_-6′ to C-1″ indicated that the *p*-hydroxyphenyl and HMG group were connected with C-1′ and C-6′ of the glucopyranosyl moiety, respectively. The anomeric proton appearing as a doublet at *δ*_H_ 4.65 with a diaxial coupling constant of 7.7 Hz suggested that the glucopyranosyl moiety must be a β-anomer. Furthermore, the NOESY correlations (Figure 3) observed between H-1′ and H-5′, between H-3′ and H-5′, and between H-2′ and H-4′ indicated that H-1′, H-3′, and H-5′ are α-oriented and H-2′ and H-4′ are β-oriented. Therefore, Compound **5** was identified as 4-hydroxyphenyl-1-*O*-[6-(hydrogen 3-hydroxy-3-methylpentanedioate)]-β-d-glucopyranoside.

The known structures of **6**–**28** were identified by a comparison of their spectroscopic data with those reported in the literatures as arbutin (**6**) [17], β-d-glucopyranosyl-(1→6)-arbutin (**7**) [18], hydroquinone (**8**) [19], 2-(4-hydroxyphenyl)propane-1,3-diol (**9**) [20], 4-methoxyphenylacetic acid (**10**) [21], 4-hydroxybenzoic acid (**11**) [22], protocatechuic acid (**12**) [22], vanillic acid (**13**) [22], gallic acid (**14**) [23], methyl gallate (**15**) [24], 1-*O*-galloyl-β-d-glucose (1**6**) [25], 3,4,8,9,10-pentahydroxydibenzo[*b*,*d*]pyran-6-one (**17**) [26], corilagin (**18**) [27], astragalin (**19**) [28], kaempferol 3-*O*-rutinoside (**20**) [29], kaempferol 3-*O*-(2″-*O*-galloyl)-rutinoside (**21**) [30], quercetin (**22**) [29], isoquercitrin (**23**) [31], quercitrin (**24**) [32], quercetin 3-*O*-(2″-*O*-galloyl)-β-d-glucopyranoside (**25**) [33], quercetin 3-*O*-(6″-*O*-galloyl)-β-d-glucopyranoside (2**6**) [34], rutin (**27**) [34], and quercetin 3-*O*-(2″-*O*-galloyl)-rutinoside (**28**) [33].

### 2.2. Anti-Tobacco Mosaic Virus Activity

All the isolated compounds were tested for their inhibitory activity against TMV using the leaf-disc method at a concentration of 0.5 mM; however, only weak to moderate inhibitory activity were observed (Table 2). The IC_50_ values of arbutin (**6**), β-d-glucopyranosyl-(1→6)-arbutin (**7**), 4-methoxyphenylacetic acid (**10**), and corilagin (**18**) were determined as 0.49, 0.51, 0.27, and 0.45 mM, while the commercial antiviral agents, ningnanmycin and ribavirin, possessed an IC_50_ of 0.18 and 0.26 mM, respectively, during the test under the same condition.

## 3. Discussion

Continuous efforts have been made since the 1980s in the phytochemical and biological study of secondary metabolites from the Chinese medicinal plant *A. altissima*. Phytochemical studies have led to the characterization of quassinoids, alkaloids, lipids, coumarins, and other phenolic derivatives, of which quassinoids are the major components, with antitumor, antimalarial, antifeedant, anti-inflammatory, and other activities [2,35]. Twenty-eight compounds, including four novel structures (**1**, **2**, **4**, and **5**), were obtained from the fruit extract of *A. altissima* in our present study. Among the known structures, 12 compounds, including **3**, **6**–**10**, **15**–**16**, **20**–**21**, **25**, and **28**, were isolated from this plant for the first time.

Previous studies have revealed the presence of alkaloids with varying structural patterns, including indole, β-carboline, as well as canthinone types [7,8,36,37,38]. However, the nitrogenous compounds **1**–**4** obtained in our present study represent two novel types that have never been reported from the secondary metabolites of *A. altissima*. Phenylpropionamides **1**–**3** contain an α-hydroxyamide scaffold, which is present in a variety of compounds with confirmed biological activity, such as pantothenic acid (vitamin B5), the cholesterol-lowering drug bestatin, and the antibiotics amikacin and cefamandole [39]. 2-Hydroxy-*N*-(2-hydroxyphenyl)propionamide (**3**) has been previously reported to be isolated from the solid cultures of phytopathogen *Peronophythora litchii*, which is a major disease of lychee that causes twig withering, panicle shattering, fruit downfall, and rot [16]. To the best of our knowledge, this is the first report of 2-hydroxy-*N*-(2-hydroxyphenyl)propionamide (**3**) from plant secondary metabolites.

Piperidine alkaloids are among the most abundant metabolites of terrestrial plants [40]. Many of them, such as piperidine alkaloids identified from several *Prosopis* species, have been reported to possess diverse bioactivities, such as antibacterial, antifungal, and antiparasitic activities [41,42]. Recently, epidihydropinidine, the main piperidine alkaloid compound of Norway spruce (*Picea abies*), was reported to show promising antibacterial and anti-*Candida* activity [43]. 2β-Carboxyl-piperidine-4β-acetic acid methyl ester (**4**) was an unusual 2,4-disubstituted piperidine derivative of plant origin. Compounds **1**–**4** showed no potent anti-TMV activity in our present study, however, these unusual structures deserve further effort to evaluate their potential biological and pharmacological value.

Among the other known compounds obtained, corilagin (**18**) is a member of the tannin family, which has been discovered in a number of medicinal plants such as the *Phyllanthus* species. Corilagin was reported to possess diverse pharmacological activities such as antioxidative, anti-inflammatory, thrombolytic, antihypertensive, hepatoprotective, and antiatherogenic activities, as well as anti-tumor action in hepatocellular carcinoma, ovarian cancer, etc. [44,45]. Meanwhile, corilagin can reduce the cytotoxicity induced by human enterovirus 71 (EV71) and Coxsackie-virus A16 (CA16) on Vero cells and has been shown to protect against HSV1 encephalitis through inhibiting the TLR2 signaling pathways in vivo and in vitro [45,46]. Our present study indicated that corilagin (**18**) showed moderate antiviral activity against the positive single strand virus TMV, the type member of genus *Tobamovirus*.

## 4. Materials and Methods

### 4.1. General Experimental Procedures

IR spectra were obtained with a Thermo Scientific Nicolet iS50 FT-IR spectrometer (Thermo Scientific, Waltham, MA, USA). ^1^H- and ^13^C-NMR spectra were obtained with a Bruker AVANCE III 500 spectrometer (Bruker BioSpin, Fällanden, Switzerland) using tetramethylsilane as an internal standard. HR-ESI-MS were obtained with an Agilent 6520 Q-TOF mass spectrometer (Agilent Technologies, Santa Clara, CA, USA). Sephadex LH-20 (25–100 μm, Pharmacia Fine Chemical Co., Ltd., Uppsala, Sweden), Lichroprep RP-18 gel (40–63 μm, Merck, Darmstade, Germany), MCI gel CHP-20P (75–150 μm, Mitsubishi Chemical Co., Tokyo, Japan), Silica gel (200–300 mesh and 300–400 mesh), and Silica gel H (Qingdao Oceanic Chemical Co., Qingdao, China) were used for column chromatography. Thin-layer chromatography (TLC) was performed on glass-backed plates coated with 0.25 mm layers of Silica gel H (Qingdao Oceanic Chemical Co., Qingdao, China). Fractions were monitored by TLC and spots were visualized by heating silica gel plates sprayed with 5% H_2_SO_4_ in EtOH. All solvents and chemicals used were of analytical reagent grade (Sinopharm Chemical Reagent Co., Ltd., Shanghai, China), and water was doubly distilled before use.

### 4.2. Plant Material

The fruit of *Ailanthus altissima* was collected in Muyang City, Jiangsu Province, China, in October 2013. The plant was identified by associate Professor Chun-Mei Huang, College of Life Sciences, Fujian Agriculture and Forestry University, Fuzhou, China. A voucher specimen (sample MF131001) was deposited at the Key Laboratory of Bio-Pesticide and Chemistry-Biology, Ministry of Education, Fujian Agriculture and Forestry University.

### 4.3. Extraction, Fraction, and Isolation

The air-dried and pulverized fruit (7.5 kg) of *A. altissima* Swingle was extracted with MeOH (25 L, 3 day) at room temperature for three times. After being concentrated in vacuo, the extract (390.0 g) was suspended in water and successively partitioned with *n*-hexane, CHCl_3_, and *n*-BuOH. The *n*-BuOH extract (90.0 g) was fractionated by silica gel (300–400 mesh) column chromatography (CC) and eluted with mixtures of CHCl_3_–MeOH–H_2_O (95:5:0, 90:10:0; 80:20:2; 70:30:5; 60:40:10; 0:100:0, each 6 L) to obtain 14 fractions (Fractions 1–14).

Fraction 2 (2.4 g) was subjected to Sephadex LH-20 CC and eluted with CHCl_3_–MeOH (1:1) to give five fractions (Fractions 2a–2e). Fraction 2d (292.0 mg) was separated using RP-18 gel CC and eluted with a gradient of MeOH–H_2_O (15:85 to 100:0) and chromatographed over a silica gel column eluted with CHCl_3_–MeOH (98:2) to yield **3** (33.0 mg) and **13** (54.0 mg). Fraction 3 (3.5 g) was separated by MCI gel CC eluted with a gradient of MeOH–H_2_O (15:85 to 100:0) to afford 16 fractions (Fractions 3a–3p). Fraction 3c (86.0 mg) was chromatographed over silica gel column and eluted with CHCl_3_–MeOH (96:4) to yield **8** (65.0 mg). Fraction 3e (460.0 mg) was subjected to RP-18 gel CC and eluted with MeOH–H_2_O (30:70) to give **15** (292.0 mg). Fraction 3k (81.0 mg) was subjected to RP-18 gel CC eluted with MeOH–H_2_O (60:40) to yield **10** (8.5 mg) and **11** (67.0 mg). Faction 4 (5.4 g) was chromatographed over MCI gel column and gradiently eluted with a mixture of MeOH–H_2_O (15:85 to 100:0) to afford **12** (90.5 mg) and **22** (37.5 mg). Fraction 6 (9.3 g) was first chromatographed over MCI gel column eluted with a gradient MeOH–H_2_O (15:85 to 100:0) and then purified using silica gel CC with CHCl_3_–MeOH (95:5) as eluate to give **9** (14.1 mg). Fraction 7 (6.3 g) was submitted to MCI gel CC and eluted with a gradient MeOH–H_2_O (15:85 to 100:0), affording 10 fractions (Fractions 7a–7j). Fraction 7b (2.6 g) was chromatographed over Sephadex LH-20 column and eluted with MeOH to yield **14** (2.5 g). Fraction 7c (314 mg) was subjected to RP-18 gel CC eluted with MeOH–H_2_O (30:70) and then purified by silica gel CC eluted with CHCl_3_–MeOH (90:10), yielding **1** (142.6 mg). Fraction 8 (10.1 g) was subjected to MCI gel CC, eluted with MeOH–H_2_O (5:95 to 100:0) to afford 11 fractions (Fractions 8a–8k). Fraction 8f (1.2 g) was subjected to Sephadex LH-20 CC eluted with MeOH to yield **17** (160.7 mg). Fraction 8g (1.0 g) was subjected to RP-18 gel CC eluted with MeOH–H_2_O (45:55), yielding **19** (50.8 mg), **23** (199.1 mg), and **24** (20.7 mg). Fraction 9 (8.9 g) was separated by MCI gel CC eluted with a gradient MeOH–H_2_O (0:100 to 100:0), chromatographed over RP-18 gel column eluted with MeOH–H_2_O (5:95), and then purified over silica gel CC with CHCl_3_–MeOH (90:10) as eluent to give **6** (97.0 mg). Fraction 10 (10.3 g) was subjected to MCI gel CC eluted with MeOH–H_2_O (5:95 to 100:0) and then chromatographed over an RP-18 gel column eluted with MeOH–H_2_O (45:55) to give **20** (33.3 mg). Fraction 11 (11.5 g) was separated using MCI gel CC eluted with a gradient mixture of MeOH–H_2_O (5:95 to 100:0), and eight fractions (Fractions 11a–11h) were obtained. Fraction 11a (3.2 g) was subjected to RP-18 gel CC eluted with MeOH–H_2_O (20:80), which was further purified by silica gel CC eluted with CHCl_3_–MeOH–H_2_O (80:20:2), to give **4** (22.8 mg) and **16** (36.8 mg). Fraction 11b (657.0 mg) was subjected to RP-18 gel CC eluted with MeOH–H_2_O (20:80) and further purified by silica gel CC with CHCl_3_–MeOH–H_2_O (80:20:2) to give **2** (11.5 mg) and **5** (10.8 mg). Fraction 11c (611.0 mg) was subjected to Sephadex LH-20 CC and eluted with MeOH to give **18** (171.0 mg). Fraction 11f (1.7 g) was subjected to RP-18 gel CC eluted with MeOH–H_2_O (45:55), affording **25** (31.3 mg), **26** (41.6 mg), and **27** (464.5 mg). Fraction 12 (7.8 g) was submitted to MCI gel CC eluted with a gradient mixture of MeOH–H_2_O (0:100 to 100:0), providing 11 fractions (Fractions 12a–12k). Fraction 12i (256.0 mg) and Fraction 12j (410.0 mg) were subjected to silica gel CC eluted with CHCl_3_–MeOH–H_2_O (70:30:5) followed by RP-18 gel CC with MeOH–H_2_O (45:55) as eluent to afford **28** (37.0 mg) and **21** (8.4 mg), respectively. Fraction 14 (1.1 g) was gradiently eluted with a mixture of MeOH–H_2_O (0:100 to 100:0) over MCI gel CC and then purified using silica gel CC with a mixture of EtOAc–acetone–MeOH (70:20:10) as eluent to afford **7** (21.7 mg).

*2-Hydroxy-N-[(2-O-β*-*d*-*glucopyranosyl)phenyl]propionamide* (**1**). White amorphous powder. IR (KBr) υ_max_: 3478, 3388, 2907, 1674, 1603, 1534, 1456, 1256, 1118, 1074, 1023 cm^−1^; HR-ESI-MS *m*/*z*: 366.1185 (Calcd. for C_15_H_21_NO_8_Na, 366.1159); ^1^H-NMR (500 MHz, DMSO-*d*_6_) and ^13^C-NMR (125 MHz, DMSO-*d*_6_) data (see Table 1).

*2-Hydroxy-N-[(2-O-β*-*d*-*glucopyranosyl-(1→6)-β*-*d*-*glucopyranosyl) phenyl]propionamide* (**2**). White amorphous powder. IR (KBr) υ_max_: 3423, 3346, 2928, 2891, 1662, 1602, 1532, 1454, 1260, 1070 cm^−1^; HR-ESI-MS *m*/*z*: 528.1726 (Calcd. for C_21_H_31_NO_13_Na, 528.1688); ^1^H-NMR (500 MHz, DMSO-*d*_6_) and ^13^C-NMR (125 MHz, DMSO-*d*_6_) data (see Table 1).

*2-Hydroxy-N-(2-hydroxyphenyl)propionamide* (**3**). White amorphous powder. HR-ESI-MS *m*/*z*: 182.0840 (Calcd. for C_9_H_12_NO_3_Na, 182.0812); ^1^H-NMR (DMSO-*d*_6_, 500 MHz) *δ* 10.06 (1H, s, OH-2′), 9.25 (1H, s, NH), 8.17 (1H, dd, *J* = 7.9, 1.3 Hz), 6.89 (2H, overlap, H-3′ and H-4′), 6.77 (1H, ddd, *J* = 7.9, 6.3, 2.5 Hz), 6.11 (1H, d, *J* = 4.9 Hz, OH-2), 4.14 (1H, qd, *J* = 6.8, 4.8 Hz, H-2), 1.32 (3H, d, *J* = 6.8 Hz). ^13^C-NMR (DMSO-*d*_6_, 125 MHz) *δ* 172.7 (C-1), 146.1 (C-2′), 126.2 (C-1′), 123.6 (C-4′), 119.1 (C-5′), 118.9 (C-6′), 114.7 (C-3′), 67.7 (C-2), 20.8 (C-3).

*2β*-*Carboxyl-piperidine-4β*-*acetic acid methyl ester* (**4**). White amorphous powder. IR (KBr) υ_max_: 3428, 2958, 1729, 1624, 1439, 1402, 1299, 1216, 1180, 1137, 1010 cm^−1^; HR-ESI-MS *m*/*z*: 224.0907 (Calcd. for C_9_H_15_NO_4_Na, 224.0899); ^1^H-NMR (methanol-*d*_4_, 500 MHz) *δ* 3.66 (3H, s, H-OC*H*_3_), 3.47 (1H, dd, *J* = 12.8, 3.2 Hz, H-2), 3.34 (1H, ddd, *J* = 12.8, 4.4, 2.2 Hz, H-6), 2.97 (1H, td, *J* = 13.2, 3.2 Hz, H-6), 2.34 (2H, d, *J* = 7.0 Hz, H-8), 2.34 (1H, overlap, H-3), 2.11 (1H, m, H-4), 1.90 (1H, brd, *J* = 14.1 Hz, H-5), 1.41 (1H, m, H-5), 1.32 (1H, dt, *J* = 14.1, 12.4 Hz, H-3). ^13^C-NMR (methanol-*d*_4_, 125 MHz) *δ* 173.8 (C-9), 173.7 (C-7), 60.6 (C-2), 52.1 (C-O*C*H_3_), 44.3 (C-6), 40.9 (C-8), 34.0 (C-3), 32.9 (C-4), 29.1 (C-5).

*4-Hydroxyphenyl-1-O-[6-(hydrogen 3-hydroxy-3-methylpentanedioate)]-β-d-glucopyranoside* (**5**). Pale-yellow solid. IR (KBr) υ_max_: 3420, 1718, 1510, 1399, 1216, 1073, 1042 cm^−1^; HR-ESI-MS *m*/*z*: 439.1234 (Calcd. for C_18_H_24_O_11_Na, 439.1211); ^1^H-NMR (DMSO-*d*_6_, 500 MHz) *δ* 6.84 (2H, d, *J* = 9.0 Hz, H-2 and H-6), 6.66 (2H, d, *J* = 9.0 Hz, H-3 and H-5), 4.65 (1H, d, *J* = 7.7 Hz, H-1′), 4.32 (1H, dd, *J* = 11.8, 2.0 Hz, H-6′), 4.00 (1H, dd, *J* = 11.8, 7.0 Hz, H-6′), 3.50 (1H, ddd, *J* = 9.2, 7.0, 2.0 Hz, H-5′), 3.26 (1H, t, *J* = 8.8 Hz, H-3′), 3.19 (1H, dd, *J* = 8.8, 7.7 Hz, H-2′), 3.14 (1H, t, *J* = 9.2 Hz, H-4′), 2.57 (1H, d, *J* = 14.0 Hz, H-2″), 2.49 (1H, overlap, H-2″), 2.40 (1H, d, *J* = 15.0 Hz, H-4″), 2.33 (1H, d, *J* = 15.0 Hz, H-4″), 1.22 (3H, s, H-6″). ^13^C-NMR (DMSO-*d*_6_, 125 MHz) *δ* 174.4 (C-5″), 170.5 (C-1″), 152.3 (C-4), 150.1 (C-1), 117.7 (C-2 and C-6), 115.5 (C-3 and C-5), 101.6 (C-1′), 76.3 (C-3′), 73.7 (C-5′), 73.2 (C-2′), 70.0 (C-4′), 68.9 (C-3″), 63.4 (C-6′), 46.1 (C-4″), 45.9 (C-2″), 27.6 (C-6″). 

### 4.4. Acid Hydrolysis

Compounds **1**, **2**, and **5** (2 mg each) were hydrolyzed in 1 M HCl (dioxane–H_2_O, 1:1, 2 mL) at 95 °C for 2 h, respectively. After evaporated to dryness, the reaction mixtures were diluted in H_2_O and extracted with Et_2_O (3 × 2 mL). The aqueous layer was neutralized with NaHCO_3_ and evaporated under vacuum to furnish a neutral residue, from which d-glucose was identified by TLC comparison with standard sugars.

### 4.5. Anti-TMV Assay

#### 4.5.1. Virus and Host Plant

Purified TMV (strain U1) was obtained from Institute of Plant Virology, Fujian Agriculture and Forestry University, Fuzhou, China, and concentration was determined as 15 mg/mL using an ultraviolet spectrophotometer method [virus concentration = (A_260_ × dilution ration)/$E_{1\;\mathrm{cm}}^{0.1\%,\;260\;\mathrm{nm}}$]. The purified virus was kept at −20 °C and diluted to 15 μg/mL with 0.01 M PBS before use. *Nicotiana tabacum* cv. K326, which were cultivated and grown to a 5–6-leaf stage in an insect-free greenhouse, were used for anti-TMV assay. 

#### 4.5.2. The Leaf-Disc Method

Pure compounds were dissolved in DMSO and diluted with 0.01 M PBS to a certain concentration for test. The final concentration of DMSO in the test solution (≤2%) showed no adverse effect on the plants. Anti-TMV assay was carried out using the leaf-disc method as described previously in our paper [47]. Growing leaves of *N. tabacum* cv. K326 were mechanically inoculated with TMV (15 μg/mL in 0.01 M PBS). After 6 h, three leaf discs (1 cm diameter) were punched and floated on solutions for test. Discs of healthy and inoculated leaves floated on a solution of 0.01 M PBS with 2% DMSO were used as a mock and control, respectively. Ningnanmycin and ribavirin were used as agent control. Three replicates were carried out for each sample. After incubating for 48 h at 25 °C in a culture chamber, the leaf discs were ground in a 0.01 M carbonate coating buffer (pH 9.6, 500 μL for each leaf disc) and centrifuged. The supernatant (200 μL) was transferred to a 96-well plate and used for an indirect enzyme-linked immunosorbent assay (ELISA). Indirect ELISA was performed as described in the literature [48,49]. Virus concentration was calculated from a standard curve constructed using OD_405_ values of purified TMV at concentrations of 1.0, 0.5, 0.25, 0.125, and 0.0625 μg/mL. The inhibition of test solutions on TMV was calculated as follows: inhibition rate = [1 − (virus concentration of treatment)/(virus concentration of control)] × 100%.

## 5. Conclusions

Two phenylpropionamides, 2-hydroxy-*N*-[(2-*O*-β-d-glucopyranosyl)phenyl]propionamide (**1**), and 2-hydroxy-*N*-[(2-*O*-β-d-glucopyranosyl-(1→6)-β-d-glucopyranosyl)phenyl]propionamide (**2**), one piperidine, 2β-carboxyl-piperidine-4β-acetic acid methyl ester (**4**), and one phenol derivative, 4-hydroxyphenyl-1-*O*-[6-(hydrogen 3-hydroxy-3-methylpentanedioate)]-β-d-glucopyranoside (**5**), as well as one known phenylpropionamide (**3**), 13 phenols (**6**–**18**), and 10 flavonoids (**19**–**28**), were identified from the *n*-BuOH-soluble fraction from MeOH extract of *Ailanthus altissima* fruit. All the compounds obtained were evaluated for their antiviral activity against TMV; however, only weak to moderate activity was observed. These results provide us with general knowledge of these diverse phenolic constituents and suggest the distribution of novel nitrogenous analogues in the metabolites of this Simaroubaceae plant.

## Figures and Tables

**Table 1:** ^1^H- (500 MHz) and ^13^C-NMR (125 MHz) data of **1** and **2** in DMSO-*d*_6_.

Position	1		2	
	*δ*_C_ (ppm)	*δ*_H_ (ppm, *J* in Hz)	*δ*_C_ (ppm)	*δ*_H_ (ppm, *J* in Hz)
1	173.1		173.2	
2	67.8	4.15 (1H, qd, 6.8, 5.1)	67.8	4.16 (1H, qd, 6.8, 5.2)
3	20.8	1.33 (3H, d, 6.8)	20.8	1.32 (1H, d, 6.8)
1′	128.6		128.0	
2′	146.5		146.4	
3′	116.4	7.20 (1H, dd, 7.5, 2.1)	116.0	7.28 (1H, dd, 8.2, 1.1)
4′	123.8	7.05 (1H, td, 7.5, 2.0)	124.1	7.05 (1H, td, 7.8, 1.8)
5′	122.6	7.02 (1H, td, 7.5, 1.8)	122.2	6.99 (1H, td, 7.7, 1.5)
6′	119.6	8.23 (1H, dd, 7.4, 2.3)	119.6	8.19 (1H, dd, 8.0, 1.8)
Glc-1″	102.1	4.85 (1H, d, 7.5)	101.5	4.86 (1H, d, 7.4)
2″	73.3	3.27–3.32 (1H, overlap)	73.3	3.25–3.34 (1H, overlap)
3″	76.5	3.27–3.32 (1H, overlap)	76.4	3.55–3.68 (1H, overlap)
4″	69.6	3.18 (1H, td, 9.2, 5.4)	69.7	3.18 (1H, td, 8.7, 5.3)
5″	77.1	3.27–3.32 (1H, overlap)	76.0	3.25–3.34 (1H, overlap)
6″	60.7	3.69 (1H, ddd, 11.8, 5.4, 2.2)	68.3	4.00 (1H, d, 10.3)
		3.49 (1H, dt, 11.8, 5.9)		3.55–3.68 (1H, overlap)
Glc-1‴			103.2	4.22 (1H, d, 7.8)
2‴			73.6	2.96 (1H, td, 8.3, 4.5)
3‴			76.6	3.10 (1H, td, 8.4, 4.5)
4‴			70.1	2.98–3.06 (1H, overlap)
5‴			76.8	2.98–3.06 (1H, overlap)
6‴			61.0	3.65 (ddd, 1H, 11.4, 5.8, 1.9)
				3.41 (1H, dt, 11.4, 5.8)
NH		9.42 (1H, s)		9.36 (1H, s)
OH-2		5.99 (1H, d, 5.1)		5.89 (1H, d, 5.2)
OH-6″		4.58 (1H, t, 5.9)		
OH-6‴				4.46 (1H, t, 5.8)

**Table 2:** Inhibitory activity against *Tobacco mosaic virus* (TMV) of **1**–**28**.

Compounds	Inhibitory Rate (%, Mean Value ± SD)	Compounds	Inhibitory Rate (%, Mean Value ± SD)
**1**	12.2 ± 2.2	**15**	–
**2**	10.8 ± 2.5	**16**	–
**3**	24.6 ± 3.5	**17**	14.0 ± 4.3
**4**	42.0 ± 4.5	**18**	57.5 ± 4.4
**5**	–	**19**	–
**6**	50.5 ± 4.3	**20**	18.0 ± 4.8
**7**	49.8 ± 4.9	**21**	38.0 ± 6.5
**8**	47.0 ± 2.6	**22**	31.9 ± 6.3
**9**	12.7 ± 2.4	**23**	–
**10**	72.3 ± 4.9	**24**	13.2 ± 6.9
**11**	–	**25**	32.3 ± 5.3
**12**	43.2 ± 5.7	**26**	37.4 ± 5.5
**13**	16.3 ± 4.9	**27**	28.4 ± 1.9
**14**	37.0 ± 5.8	**28**	45.0 ± 6.3
Ningnanmycin	86.9 ± 3.6	Ribavirin	73.9 ± 4.5

– No inhibitory effect observed.

## Supplementary Materials

The following are available online. HRESIMS, ^1^H- and ^13^C-NMR spectra of **1**–**5**; IR, DEPT, COSY, HSQC, HMBC, and NOESY of **1**, **2**, **4**, **5**; ^1^H- and ^13^C-NMR data of **6**–**28**.
