# Supplementary material for: Phenylpropionamides, Piperidine, and Phenolic Derivatives from the Fruit of Ailanthus altissima
Source: Molecules. 2017 Dec 4;22(12):2107. doi: 10.3390/molecules22122107 (PMC6149757; doi:10.3390/molecules22122107)
Supplement: Supplementary file 1 [file molecules-22-02107-s001.zip › Supplementary Materials.pdf]

Supplementary Materials

Title: Phenylpropionamides, Piperidine and Phenolic Derivatives from the Fruit of *Ailanthus altissima*

Authors: Jian-Cheng Ni, Jian-Ting Shi, Qing-Wei Tan*, and Qi-Jian Chen*

S1. IR spectra of 2-hydroxy-*N*-[(2-*O*-β-D-glucopyranosyl)phenyl]propionamide (1).

S2. HRESIMS spectra of 2-hydroxy-*N*-[(2-*O*-β-D-glucopyranosyl)phenyl]propionamide (1).

S3. ^{1}H-NMR spectra of 2-hydroxy-*N*-[(2-*O*-β-D-glucopyranosyl)phenyl]propionamide (1) (500 MHz, DMSO-d_6).

S4. ^{13}C NMR spectra of 2-hydroxy-*N*-[(2-*O*-β-D-glucopyranosyl)phenyl]propionamide (1) (125 MHz, DMSO-d_6).

S5. DEPT of 2-hydroxy-*N*-[(2-*O*-β-D-glucopyranosyl)phenyl]propionamide (1).

S6. ^{1}H-^{1}H COSY of 2-hydroxy-*N*-[(2-*O*-β-D-glucopyranosyl)phenyl]propionamide (1).

S7. HSQC of 2-hydroxy-*N*-[(2-*O*-β-D-glucopyranosyl)phenyl]propionamide (1).

S8. HMBC of 2-hydroxy-*N*-[(2-*O*-β-D-glucopyranosyl)phenyl]propionamide (1).

S9. NOESY of 2-hydroxy-*N*-[(2-*O*-β-D-glucopyranosyl)phenyl]propionamide (1).

S10. IR spectra of 2-hydroxy-*N*-[(2-*O*-β-D-glucopyranosyl-(1→6)-β-D-glucopyranosyl)phenyl]propionamide (2).

S11. HRESIMS of 2-hydroxy-*N*-[(2-*O*-β-D-glucopyranosyl-(1→6)-β-D-glucopyranosyl)phenyl]propionamide (2).

S12. ^{1}H-NMR spectra of 2-hydroxy-*N*-[(2-*O*-β-D-glucopyranosyl-(1→6)-β-D-glucopyranosyl)phenyl]propionamide (2) (500 MHz, DMSO-d_6).

S13. ^{13}C-NMR spectra of 2-hydroxy-*N*-[(2-*O*-β-D-glucopyranosyl-(1→6)-β-D-glucopyranosyl)phenyl]propionamide (2) (125 MHz, DMSO-d_6).

S14. DEPT of 2-hydroxy-*N*-[(2-*O*-β-D-glucopyranosyl-(1→6)-β-D-glucopyranosyl)phenyl]propionamide (2).

S15. ^{1}H-^{1}H COSY of 2-hydroxy-*N*-[(2-*O*-β-D-glucopyranosyl-(1→6)-β-D-glucopyranosyl)phenyl]propionamide (2).

S16. HSQC of 2-hydroxy-*N*-[(2-*O*-β-D-glucopyranosyl-(1→6)-β-D-glucopyranosyl)phenyl]propionamide (2).

S17. HMBC of 2-hydroxy-*N*-[(2-*O*-β-D-glucopyranosyl-(1→6)-β-D-glucopyranosyl)phenyl]propionamide (2).

S18. NOESY of 2-hydroxy-*N*-[(2-*O*-β-D-glucopyranosyl-(1→6)-β-D-glucopyranosyl)phenyl]propionamide (2).

S19. HRESIMS of 2-hydroxy-*N*-(2-hydroxyphenyl)propionamide (3).

S20. ^{1}H-NMR spectra of 2-hydroxy-*N*-(2-hydroxyphenyl)propionamide (3) (500 MHz, DMSO-d_6).

S21. ^{13}C-NMR spectra of 2-hydroxy-*N*-(2-hydroxyphenyl)propionamide (3) (125 MHz, DMSO-d_6).

S22. IR spectra of 2β-carboxyl-piperidine-4β-acetic acid methyl ester (4).

S23. HRESIMS of 2β-carboxyl-piperidine-4β-acetic acid methyl ester (4).

S24. ^{1}H-NMR spectraof 2β-carboxyl-piperidine-4β-acetic acid methyl ester (4) (500 MHz, CD_3OD).

S25. ^{13}C-NMR spectra of 2β-carboxyl-piperidine-4β-acetic acid methyl ester (4) (125 MHz, CD_3OD).

S26. DEPT of 2β-carboxyl-piperidine-4β-acetic acid methyl ester (4).

S27. ^{1}H-^{1}H COSY of 2β-carboxyl-piperidine-4β-acetic acid methyl ester (4).

S28. HSQC of 2β-carboxyl-piperidine-4β-acetic acid methyl ester (4).

S29. HMBC of 2β-carboxyl-piperidine-4β-acetic acid methyl ester (4).

S30. NOESY of 2β-carboxyl-piperidine-4β-acetic acid methyl ester (4).

S31. IR spectra of 4-hydroxyphenyl-1-*O*-[6-(hydrogen 3-hydroxy- 3-methylpentanedioate)]-β-D-glucopyranoside (5).

S32. HRESIMS of 4-hydroxyphenyl-1-*O*-[6-(hydrogen 3-hydroxy-3-methylpentanedioate)]-β-D-glucopyranoside (5).

S33. ^{1}H-NMR spectra of 4-hydroxyphenyl-1-*O*-[6-(hydrogen 3-hydroxy-3-methylpentanedioate)]-β-D- glucopyranoside (5) (500 MHz, DMSO-d_6).

S34. ^{13}C NMR spectra of 4-hydroxyphenyl-1-*O*-[6-(hydrogen 3-hydroxy-3-methylpentanedioate)]-β-D- glucopyranoside (5) (125 MHz, DMSO-d_6).

S35. DEPT of 4-hydroxyphenyl-1-*O*-[6-(hydrogen 3-hydroxy-3-methyl pentanedioate)]-β-D-glucopyranoside (5).

S36. ^{1}H-^{1}H COSY of 4-hydroxyphenyl-1-*O*-[6-(hydrogen 3-hydroxy-3-methylpentanedioate)]-β-D-glucopyranoside (5).

S37. HSQC of 4-hydroxyphenyl-1-*O*-[6-(hydrogen 3-hydroxy-3-methylpentanedioate)]-β-D-glucopyranoside (5).

S38. HMBC of 4-hydroxyphenyl-1-*O*-[6-(hydrogen 3-hydroxy-3-methylpentanedioate)]-β-D-glucopyranoside (5).

S39. NOESY of 4-hydroxyphenyl-1-*O*-[6-(hydrogen 3-hydroxy-3-methylpentanedioate)]-β-D-glucopyranoside (5).

S40. ^{1}H- and ^{13}C-NMR data of compounds 6–10 (δ in ppm, J in Hz).

S41. ^{1}H- and ^{13}C-NMR data of compounds 11–16 (δ in ppm, J in Hz).

S42. ^{1}H- and ^{13}C-NMR data of compounds 17 and 18 (δ in ppm, J in Hz).

S43. ^{1}H- and ^{13}C-NMR data of compounds 19, 20, and 27 (δ in ppm, J in Hz).

S44. ^{1}H- and ^{13}C-NMR data of compounds 21 and 28 (δ in ppm, J in Hz).

S45. ^{1}H- and ^{13}C-NMR data of compounds 22–26 (δ in ppm, J in Hz).

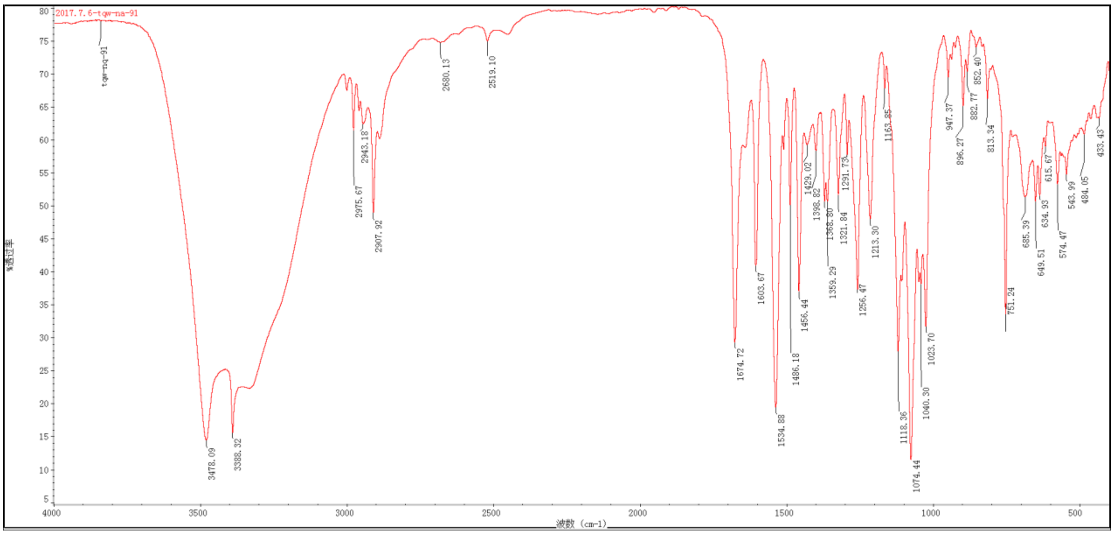

S1. IR spectra of 2-hydroxy-*N*-[(2-*O*-β-D-glucopyranosyl)phenyl]propionamide (1)

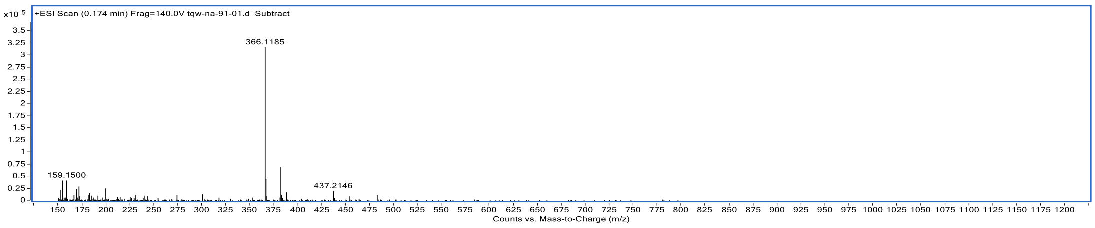

S2. HRESIMS spectra of 2-hydroxy-*N*-[(2-*O*-β-D-glucopyranosyl)phenyl]propionamide (1)

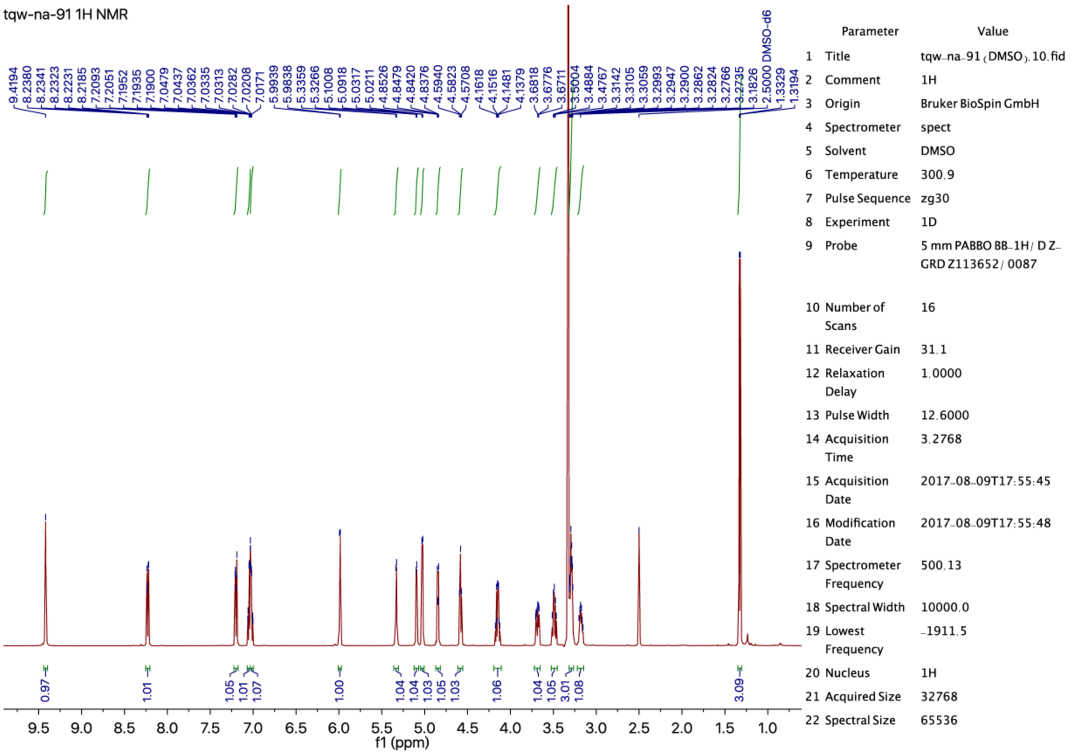

S3. ¹H-NMR spectra of 2-hydroxy-*N*-[(2-*O*-β-D-glucopyranosyl)phenyl]propionamide (1) (500 MHz, DMSO-d_6)

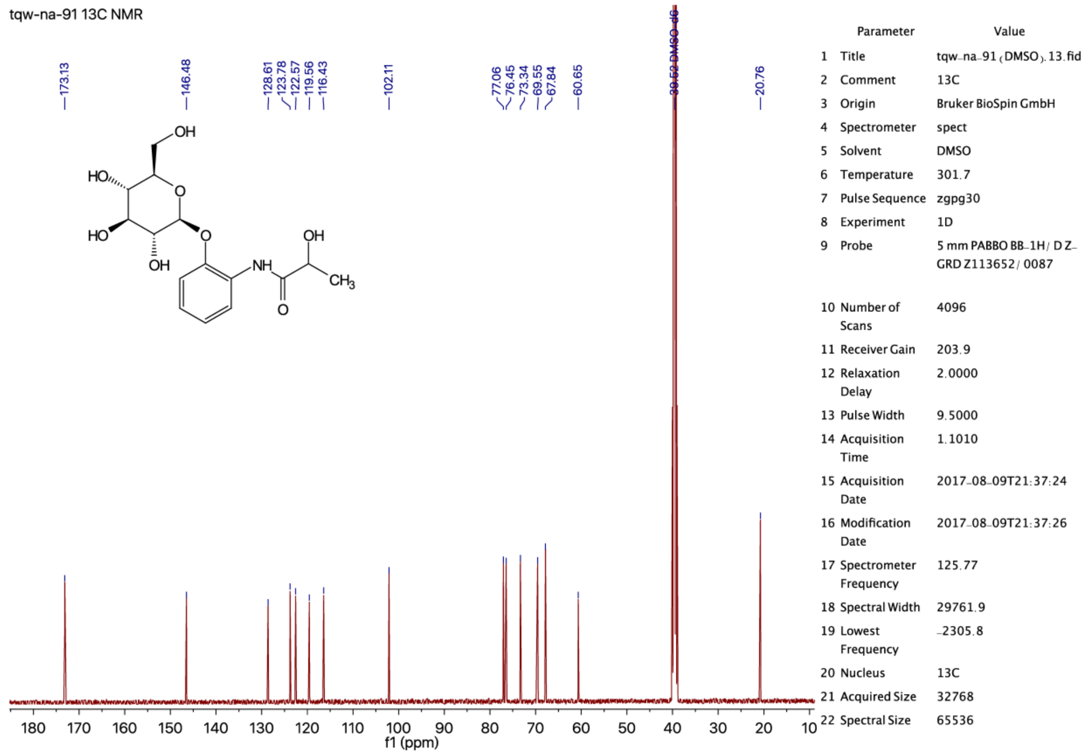

S4. **^{13}C NMR spectra of 2-hydroxy-*N*-[(2-*O*-β-D-glucopyranosyl)phenyl]propionamide (1) (125 MHz, DMSO-*d*$_6$)**

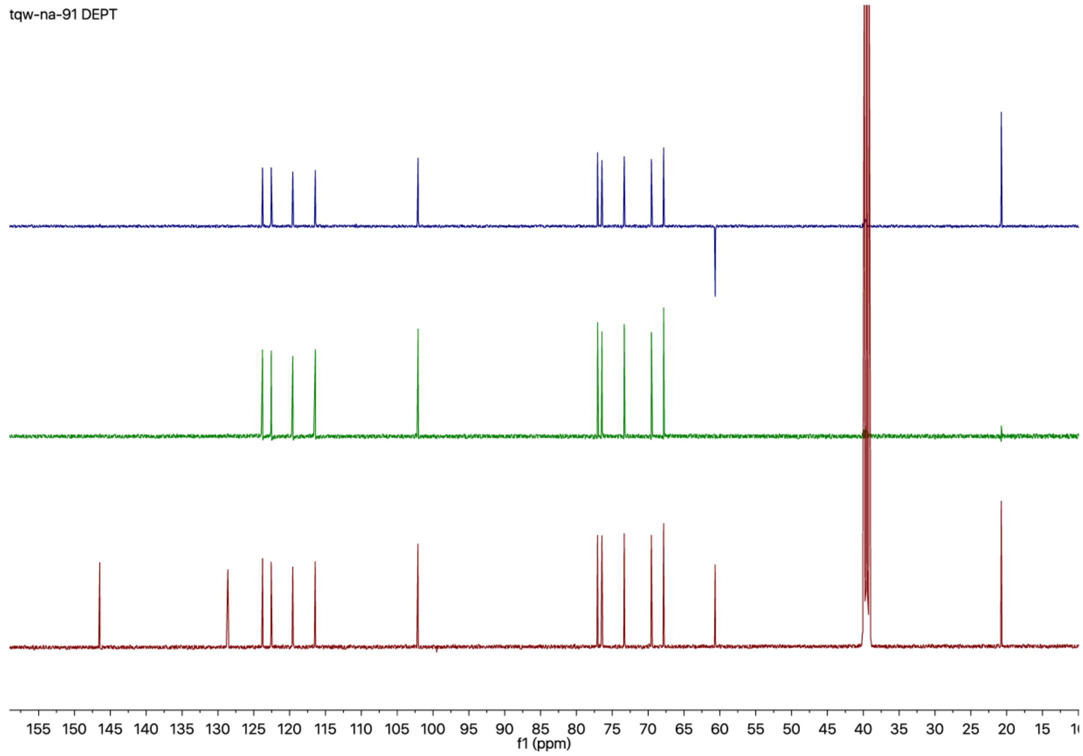

S5. DEPT of 2-hydroxy-*N*-[(2-*O*-β-D-glucopyranosyl)phenyl]propionamide (1)

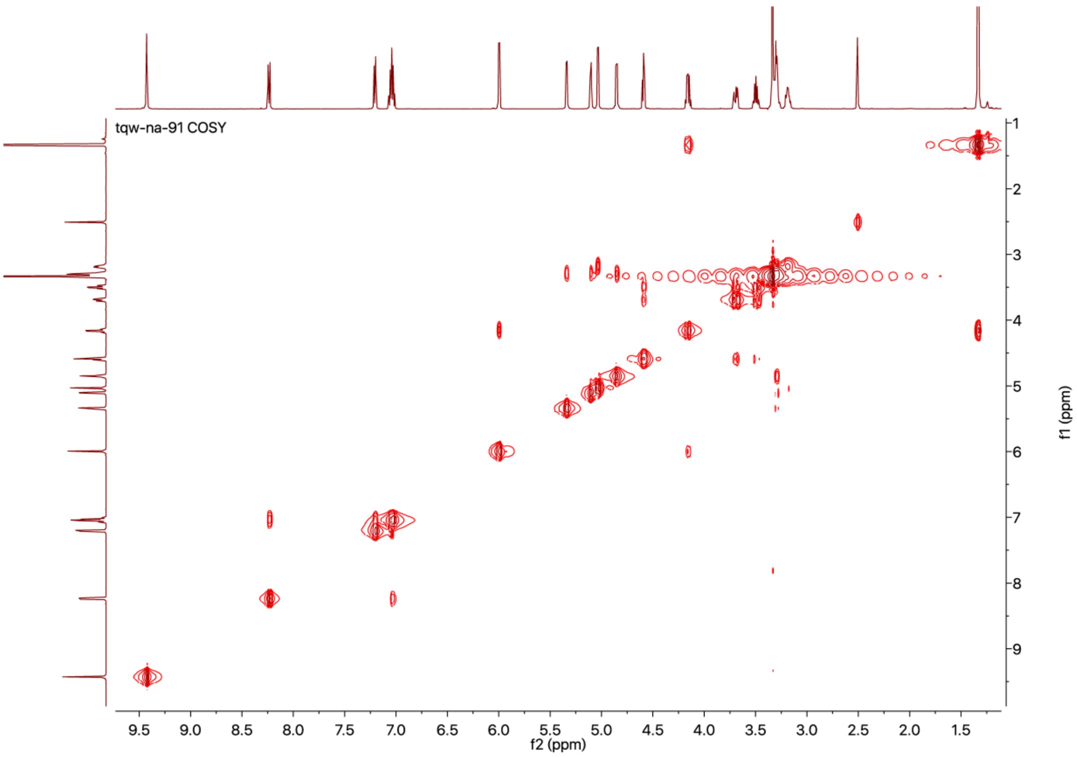

S6. ¹H-¹H COSY of 2-hydroxy-*N*-[(2-*O*-β-D-glucopyranosyl)phenyl]propionamide (1)

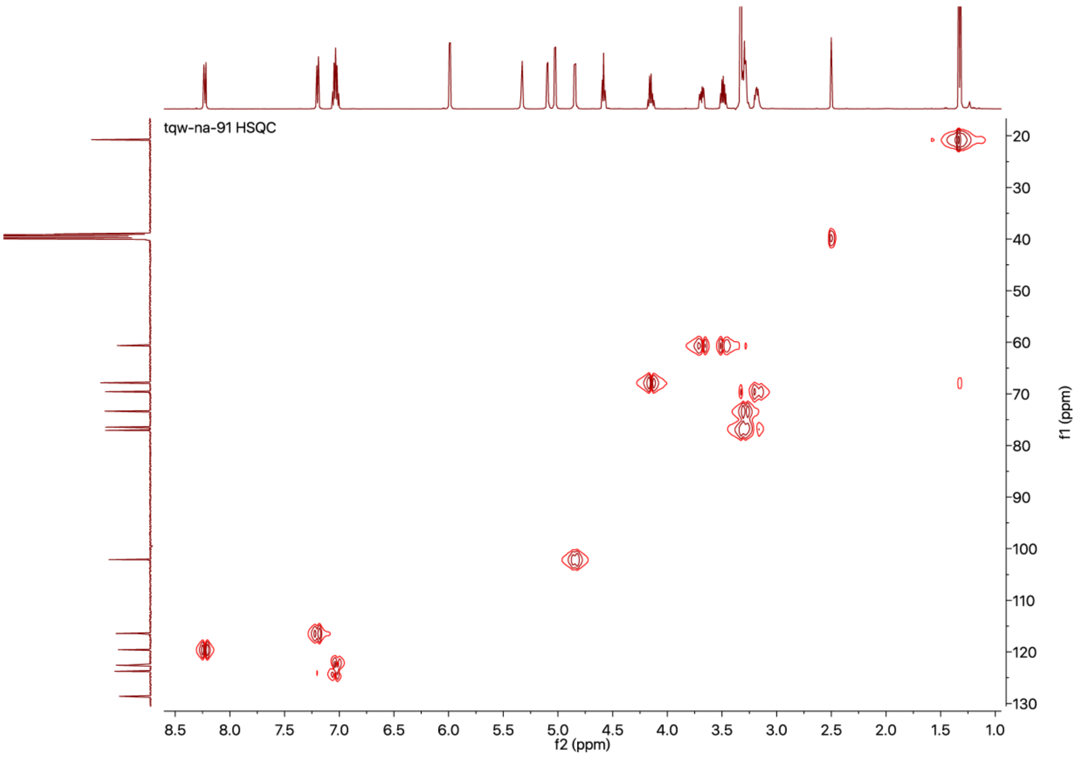

S7. HSQC of 2-hydroxy-*N*-[(2-*O*-β-D-glucopyranosyl)phenyl]propionamide (1)

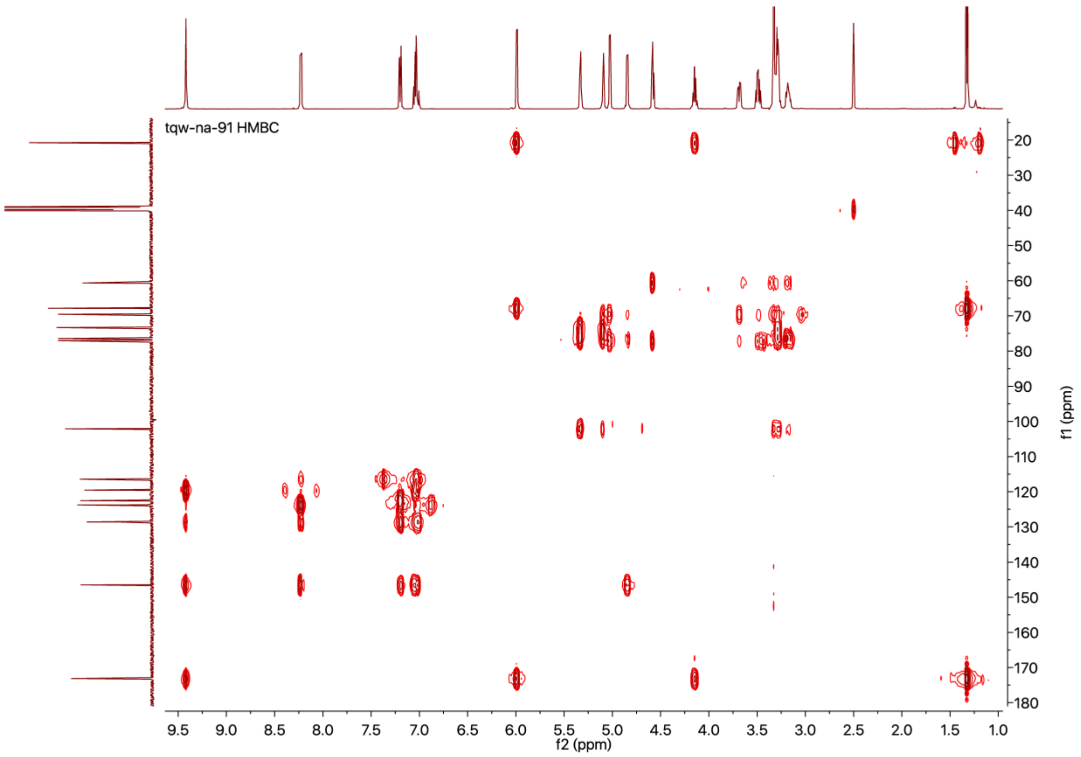

S8. HMBC of 2-hydroxy-*N*-[(2-*O*-β-D-glucopyranosyl)phenyl]propionamide (1)

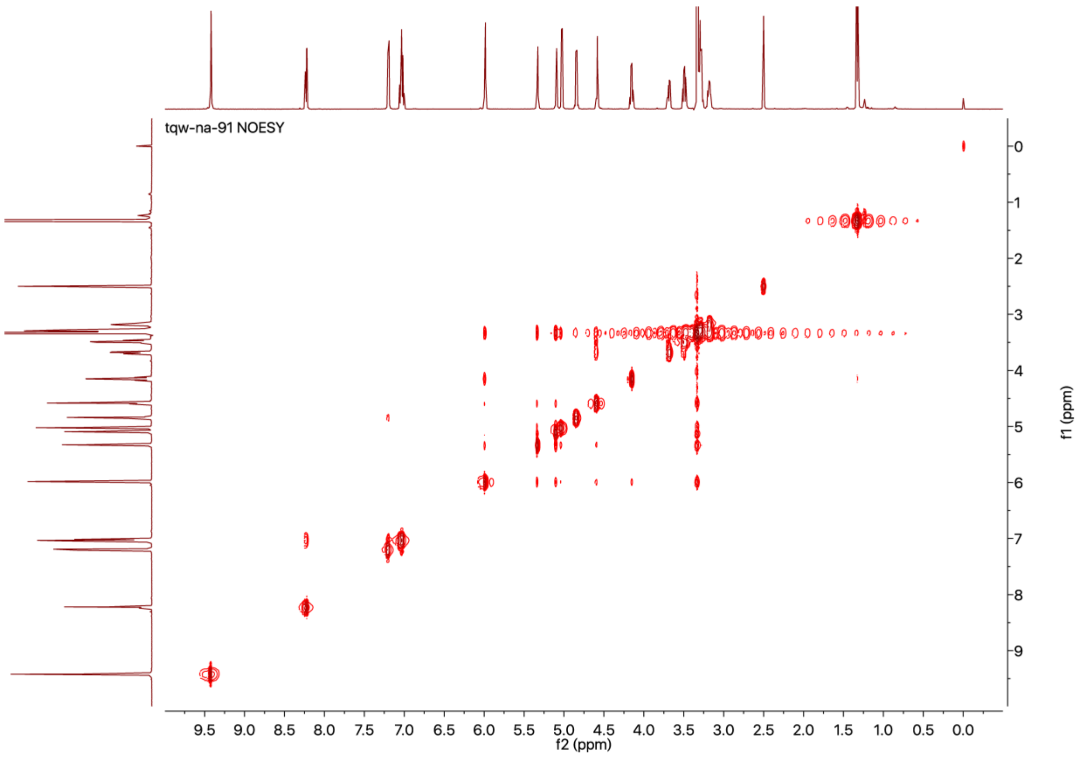

S9. NOESY of 2-hydroxy-*N*-[(2-*O*-β-D-glucopyranosyl)phenyl]propionamide (1)

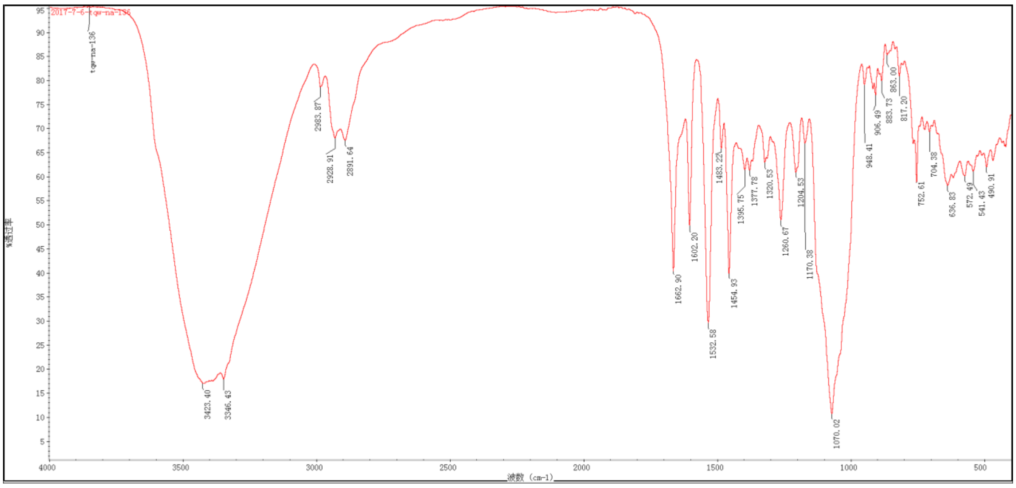

S10. IR spectra of 2-hydroxy-*N*-[(2-*O*-β-D-glucopyranosyl-(1→6)-β-D-glucopyranosyl) phenyl]propionamide (2)

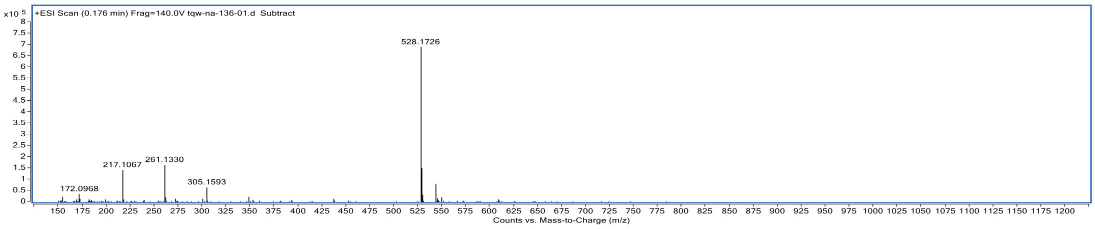

S11. HRESIMS of 2-hydroxy-*N*-[(2-*O*-β-D-glucopyranosyl-(1→6)-β-D-glucopyranosyl) phenyl]propionamide (2)

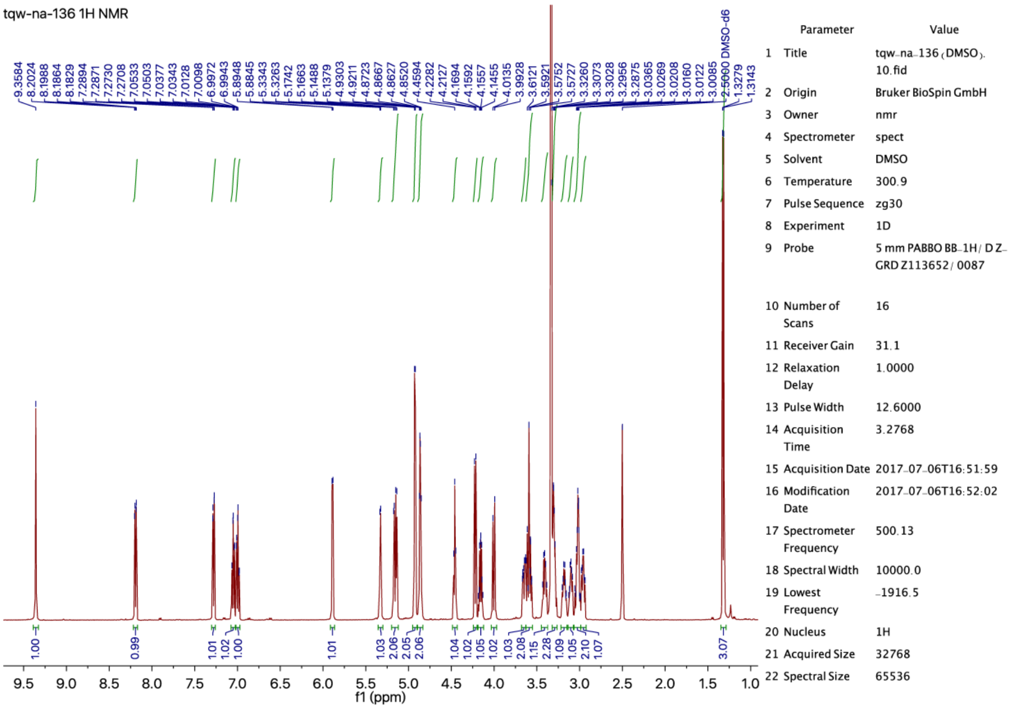

S12. ^{1}H-NMR spectra of 2-hydroxy-*N*-[(2-*O*-β-D-glucopyranosyl-(1→6)-β-D-glucopyranosyl) phenyl]propionamide (2) (500 MHz, DMSO-d_6)

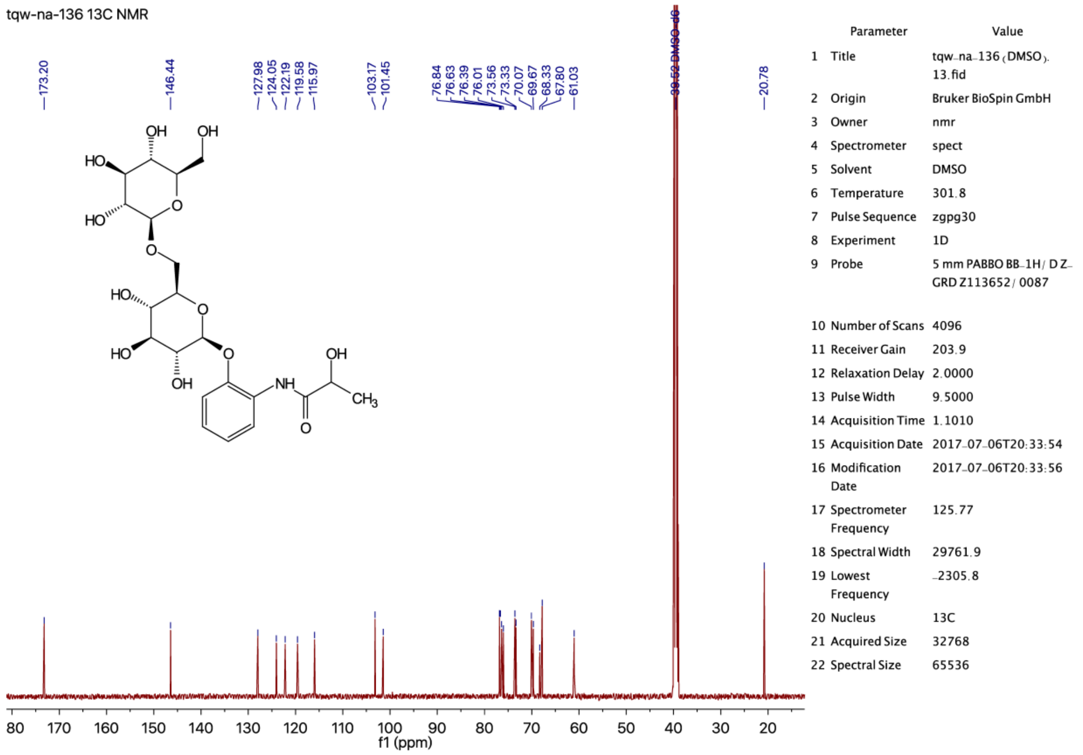

S13. ^{13}C-NMR spectra of 2-hydroxy-*N*-[(2-*O*-β-D-glucopyranosyl-(1→6)-β-D-glucopyranosyl) phenyl]propionamide (2) (125 MHz, DMSO-d_6)

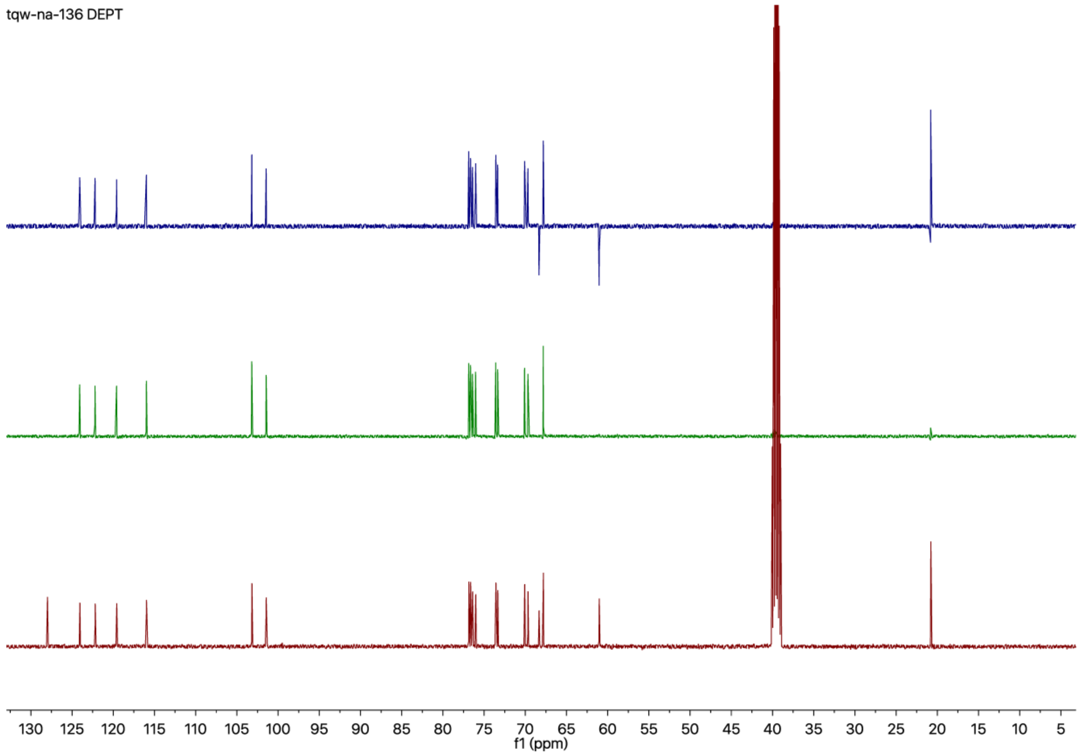

S14. DEPT of 2-hydroxy-*N*-[(2-*O*-β-D-glucopyranosyl-(1 → 6)-β-D-glucopyranosyl) phenyl]propionamide (2)

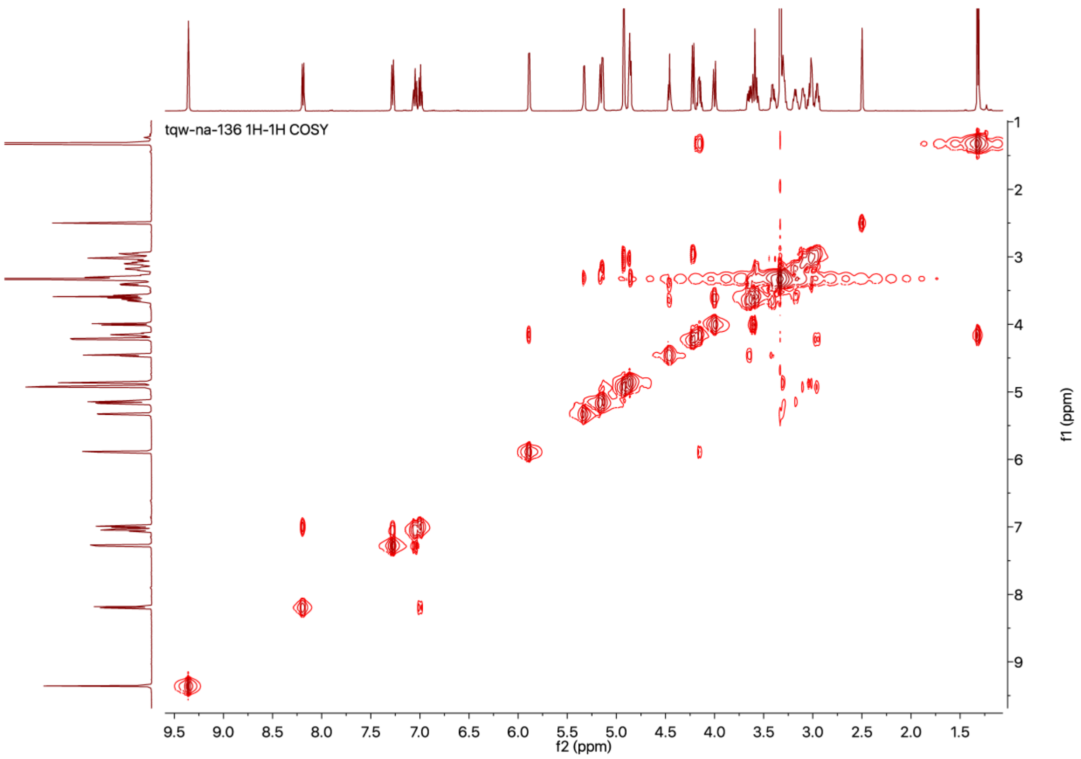

S15. ^{1}H-^{1}H **COSY of 2-hydroxy-*N*-[(2-*O*-β-D-glucopyranosyl-(1→6)-β-D-glucopyranosyl) phenyl]propionamide (2)**

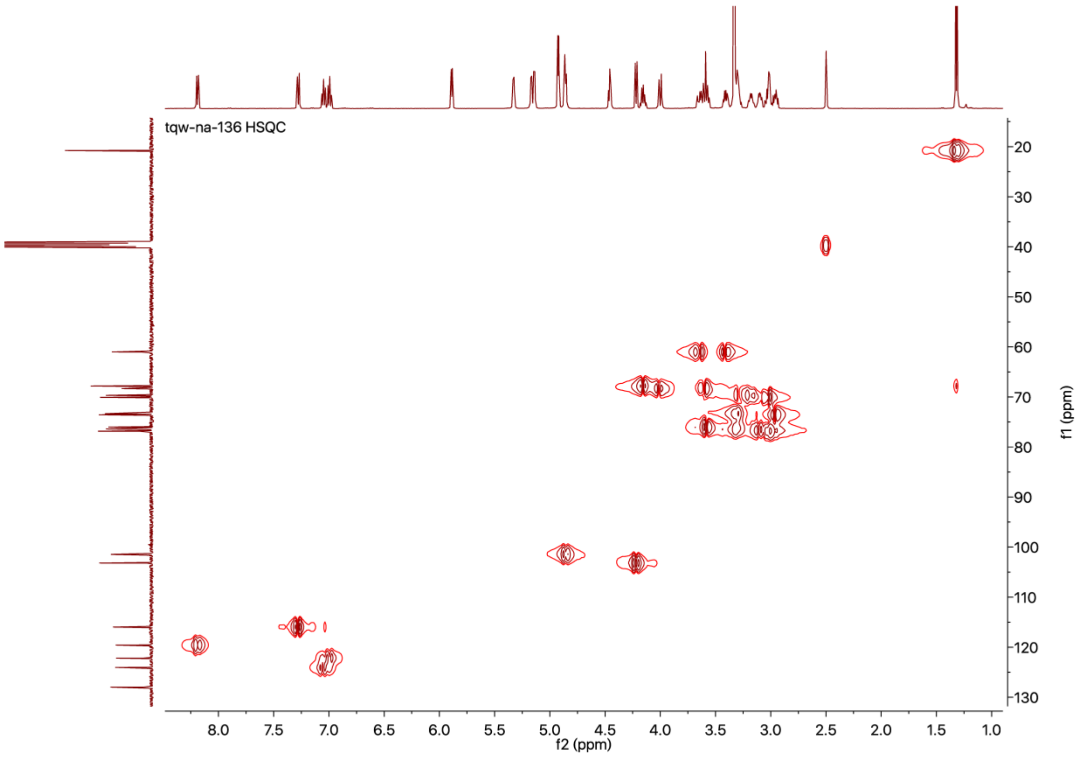

S16. HSQC of 2-hydroxy-*N*-[(2-*O*-β-D-glucopyranosyl-(1 → 6)-β-D-glucopyranosyl) phenyl]propionamide (2)

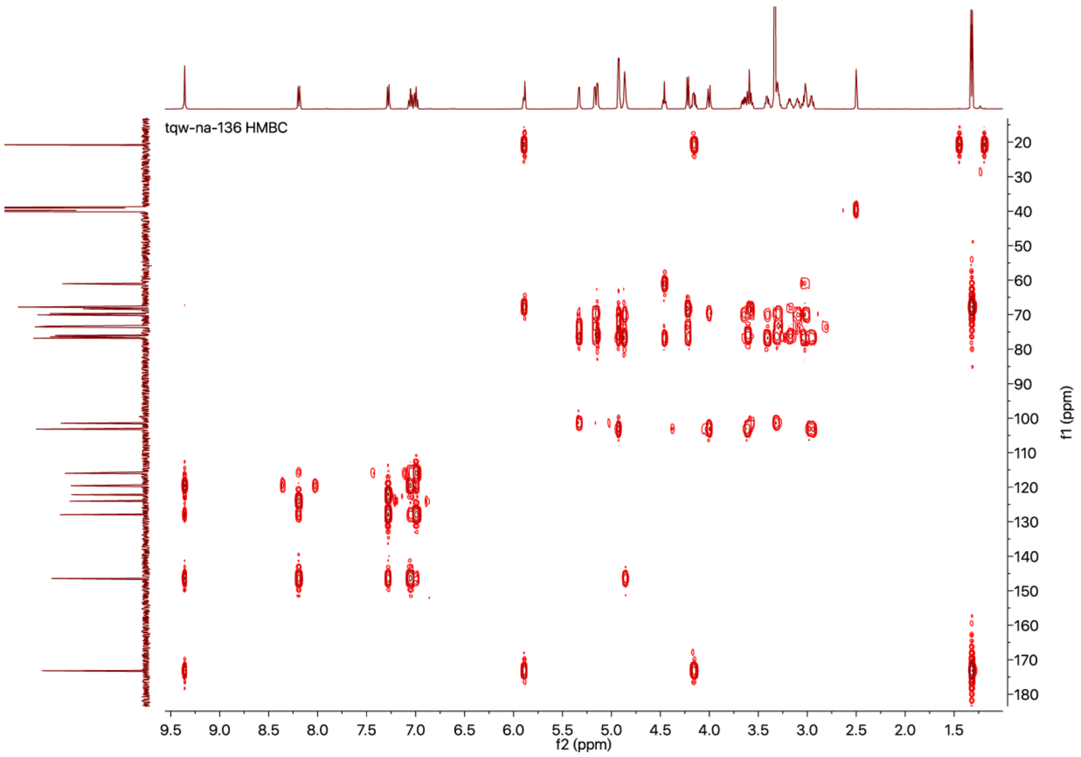

S17. HMBC of 2-hydroxy-*N*-[(2-*O*-β-D-glucopyranosyl-(1 → 6)-β-D-glucopyranosyl) phenyl]propionamide (2)

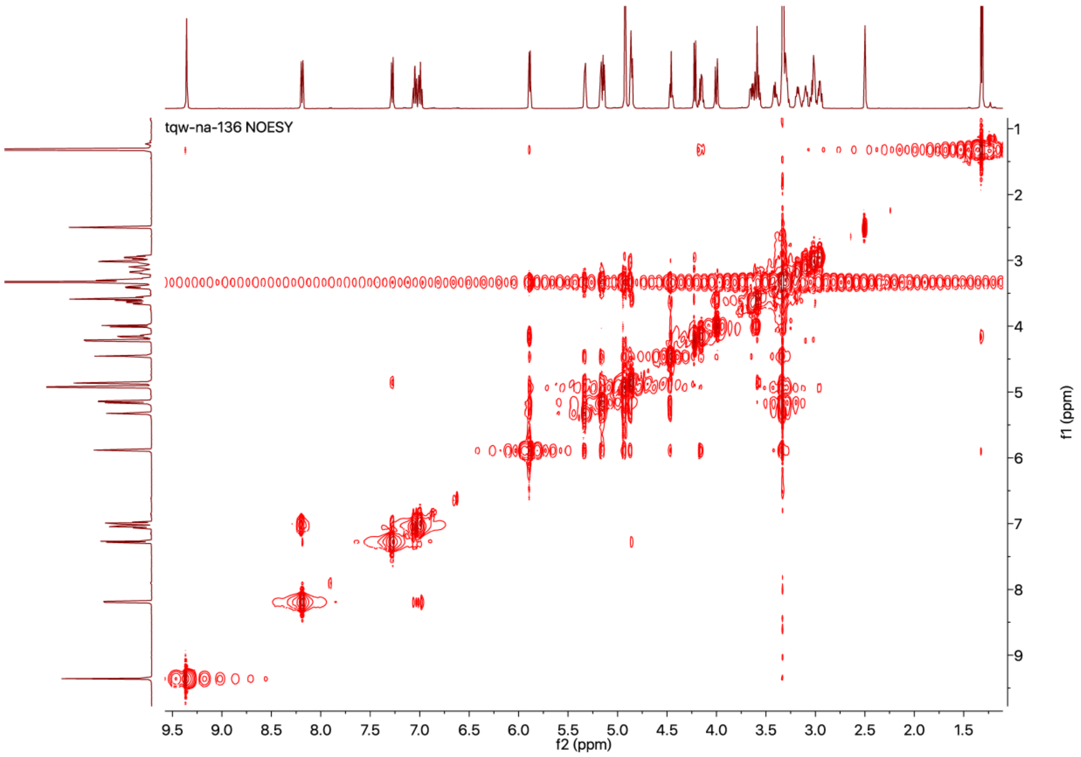

S18. NOESY of 2-hydroxy-*N*-[(2-*O*-β-D-glucopyranosyl-(1 → 6)-β-D-glucopyranosyl) phenyl]propionamide (2)

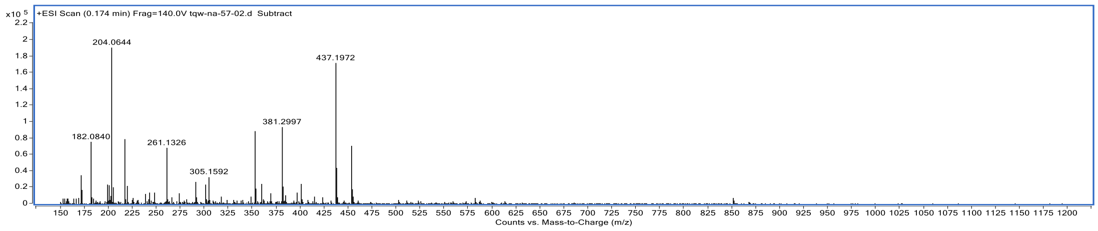

S19. HRESIMS of 2-hydroxy-*N*-(2-hydroxyphenyl)propionamide (3)

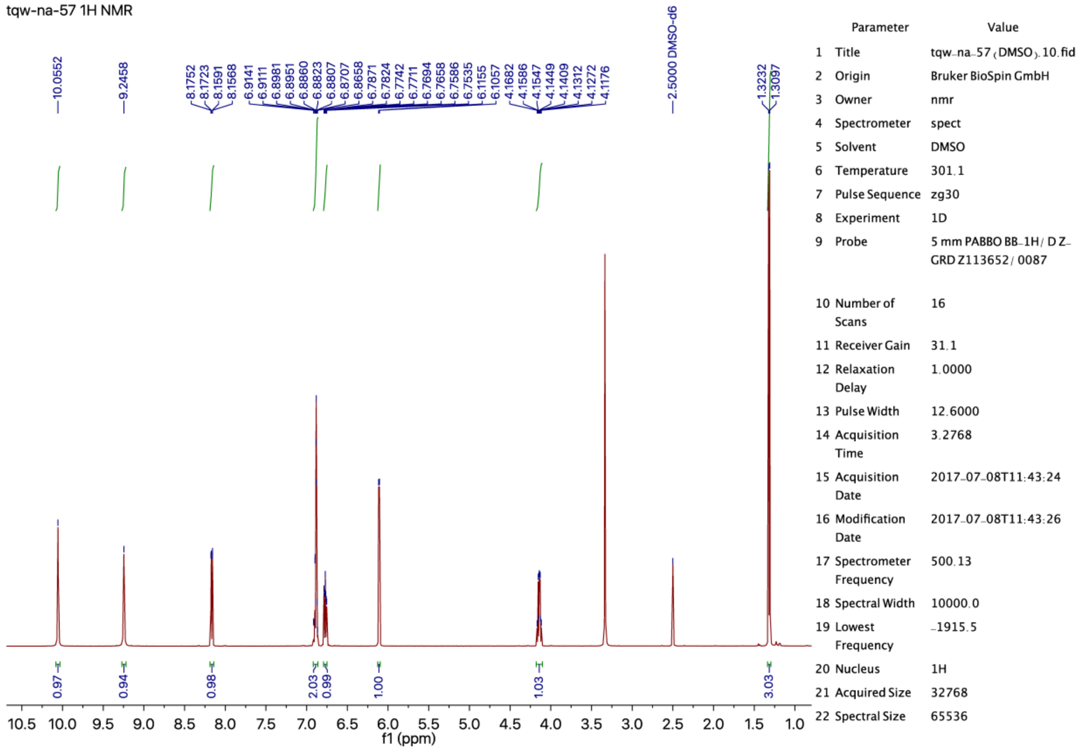

S20. ^{1}H-NMR spectra of 2-hydroxy-*N*-(2-hydroxyphenyl)propionamide (3) (500 MHz, DMSO-d_6)

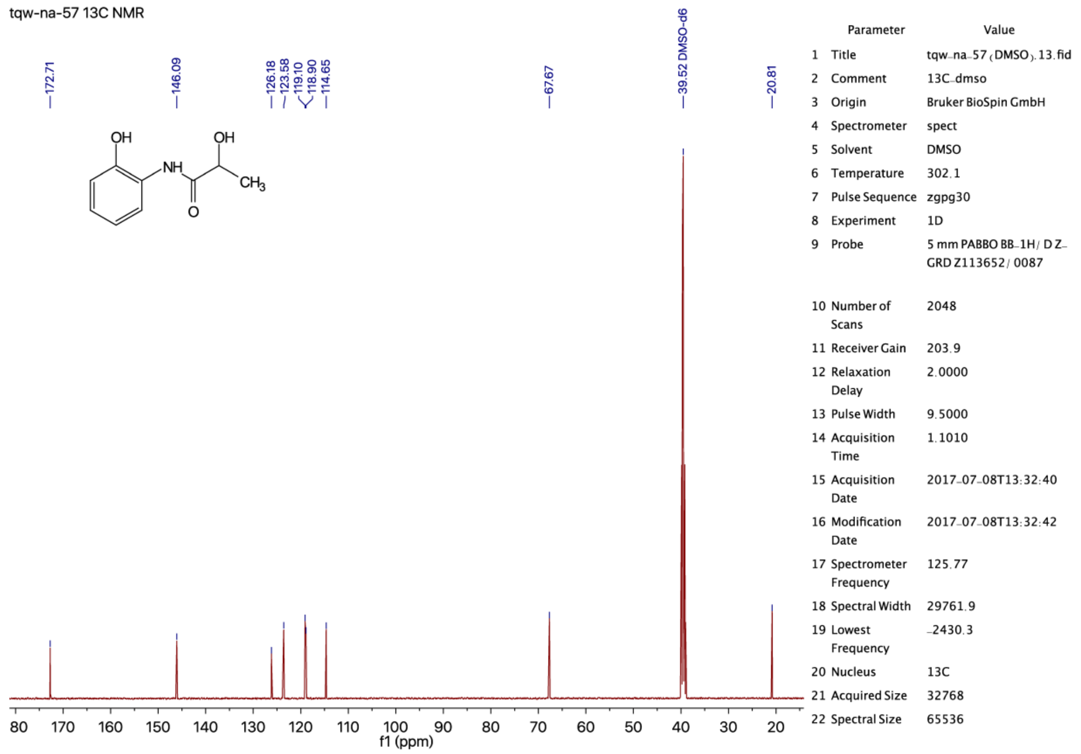

S21. ^{13}C-NMR spectra of 2-hydroxy-*N*-(2-hydroxyphenyl)propionamide (3) (125 MHz, DMSO-*d*$_6$)

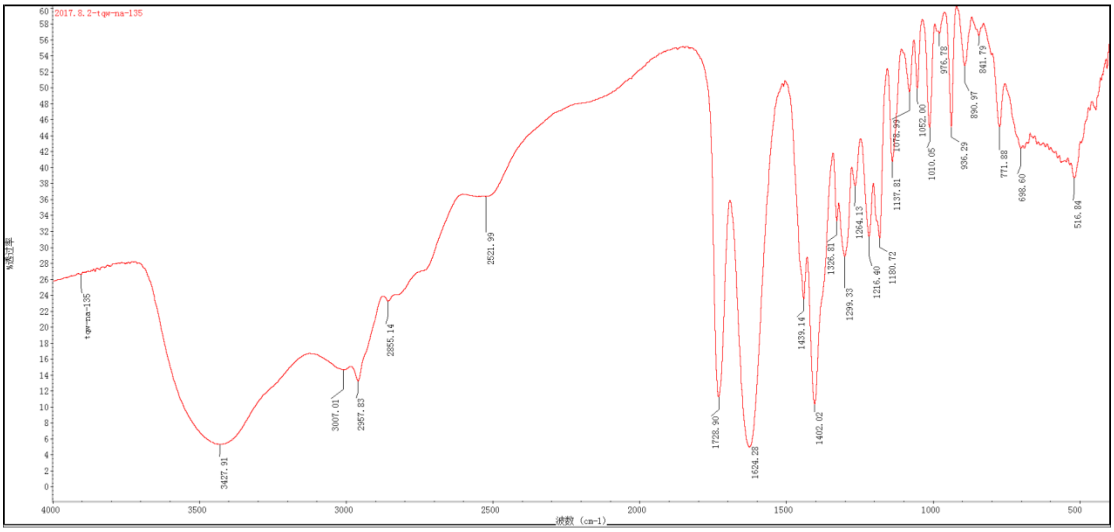

S22. IR spectra of 2β-carboxyl-piperidine-4β-acetic acid methyl ester (4)

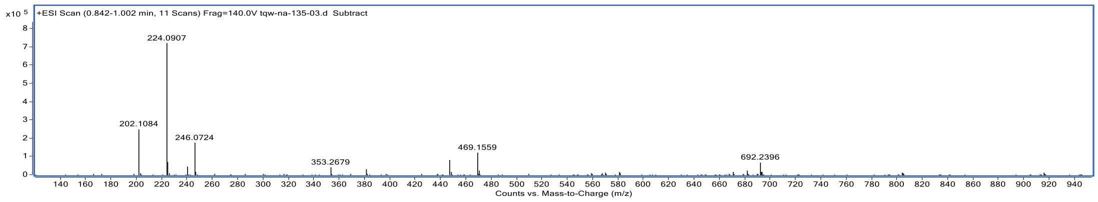

S23. HRESIMS of 2β-carboxyl-piperidine-4β-acetic acid methyl ester (4)

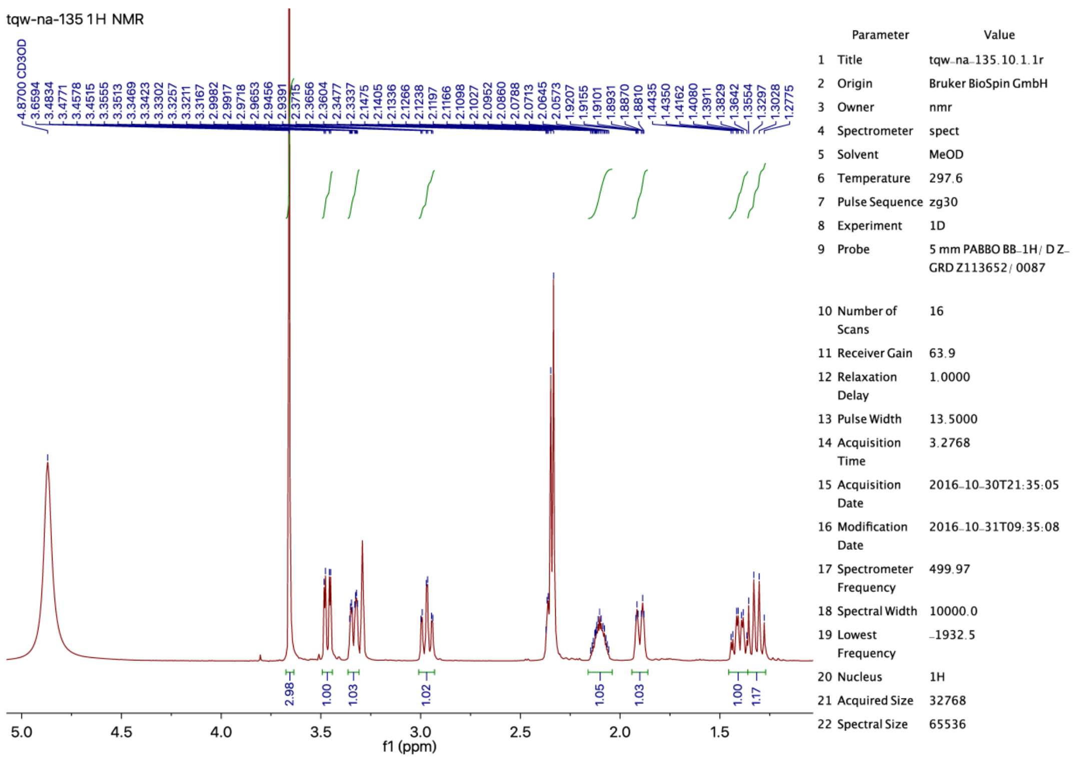

S24. **^{1}H-NMR spectraof 2β-carboxyl-piperidine-4β-acetic acid methyl ester (4) (500 MHz, CD_3OD)**

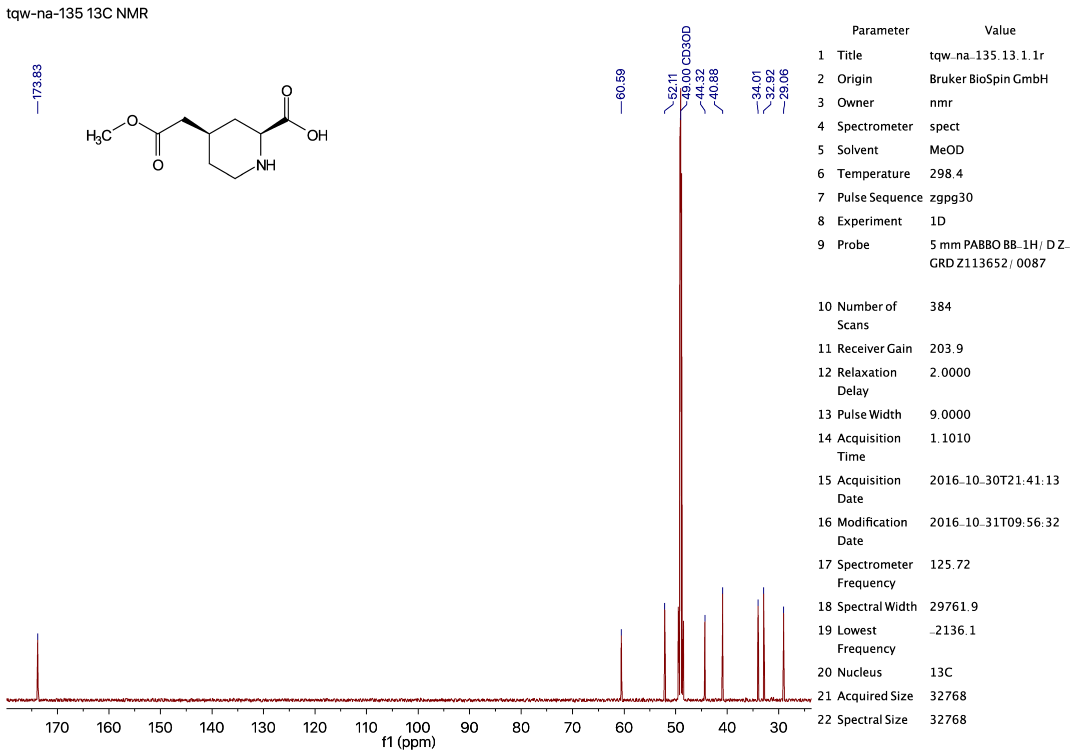

S25. **^{13}C-NMR spectra of 2β-carboxyl-piperidine-4β-acetic acid methyl ester (4) (125 MHz, CD_3OD)**

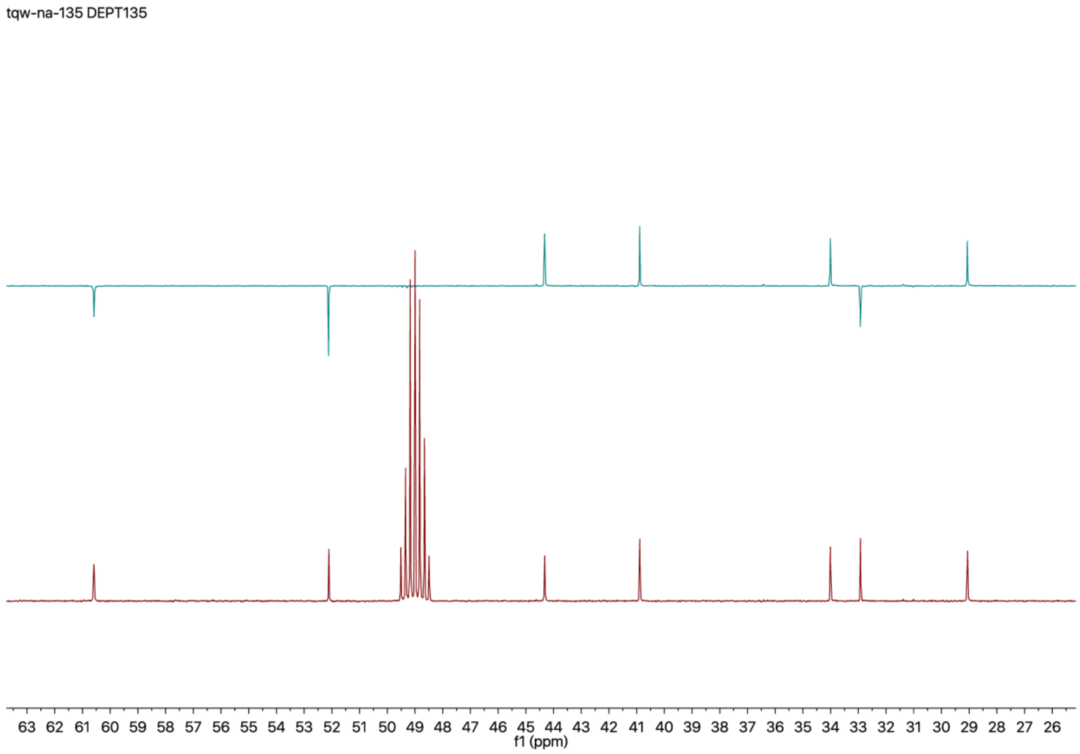

S26. DEPT of 2β-carboxyl-piperidine-4β-acetic acid methyl ester (4)

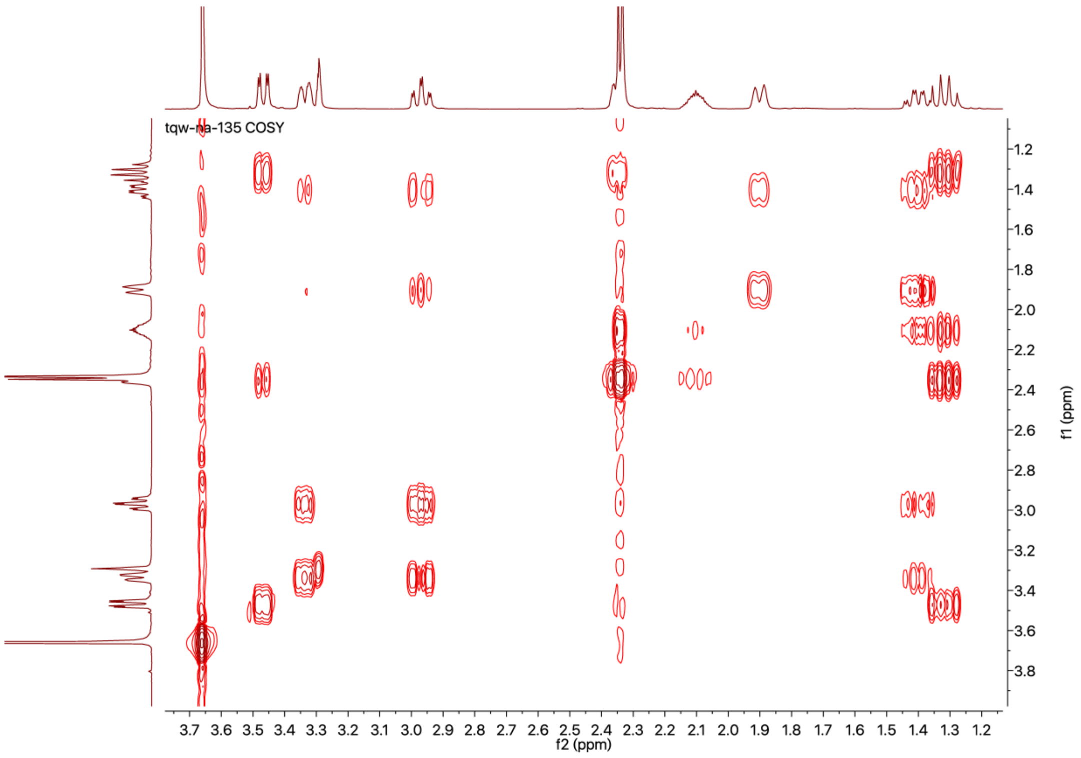

S27. ^{1}H-^{1}H COSY of 2β-carboxyl-piperidine-4β-acetic acid methyl ester (4)

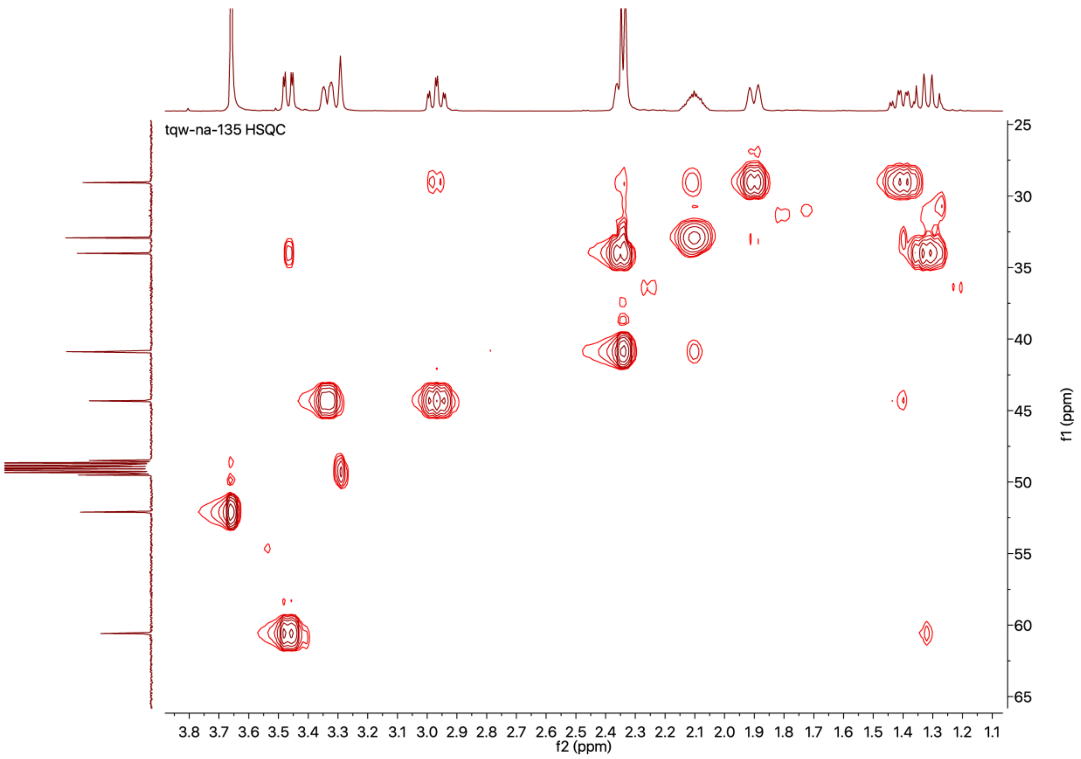

S28. HSQC of 2β-carboxyl-piperidine-4β-acetic acid methyl ester (4)

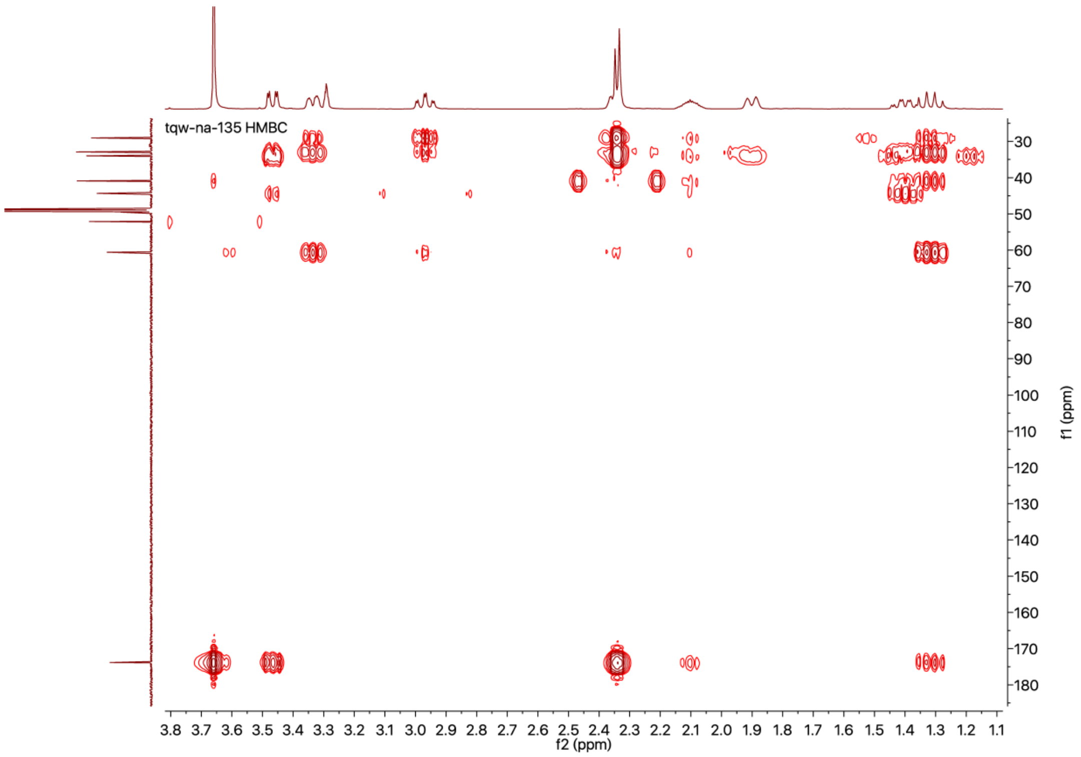

S29. HMBC of 2β-carboxyl-piperidine-4β-acetic acid methyl ester (4)

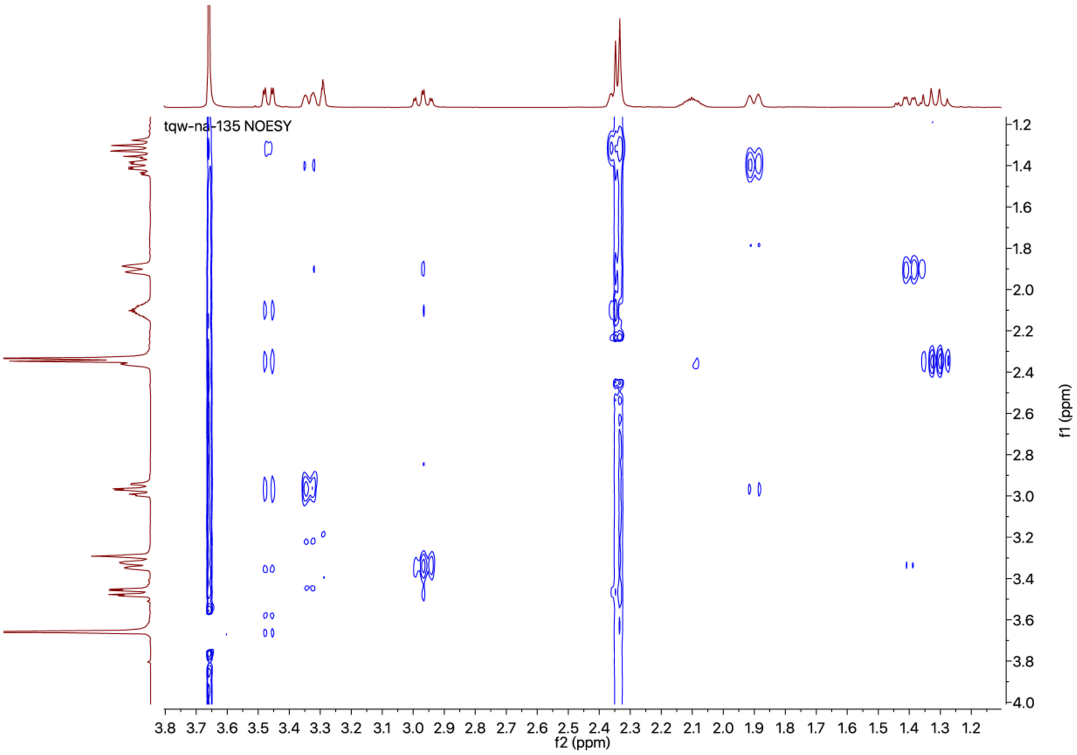

S30. NOESY of 2β-carboxyl-piperidine-4β-acetic acid methyl ester (4)

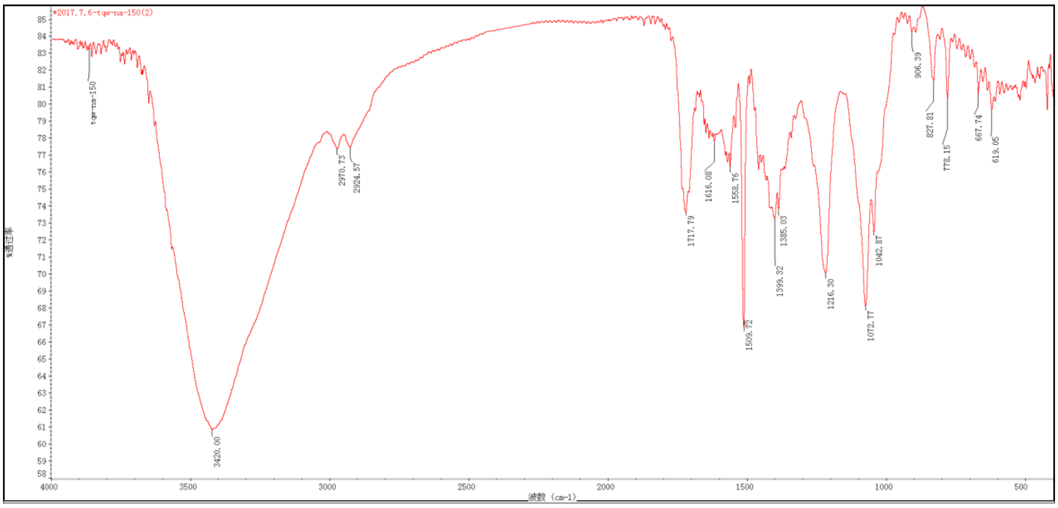

S31. IR spectra of 4-hydroxyphenyl-1-*O*-[6-(hydrogen 3-hydroxy- 3-methylpentanedioate)]-β-D-glucopyranoside (5)

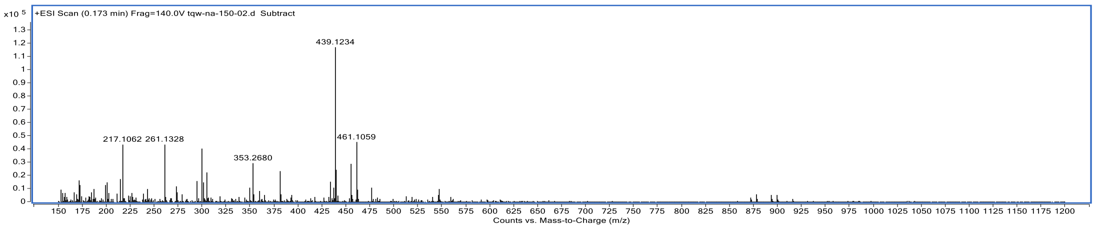

S32. HRESIMS of 4-hydroxyphenyl-1-*O*-[6-(hydrogen 3-hydroxy-3-methylpentanedioate)]-β-D-glucopyranoside (5)

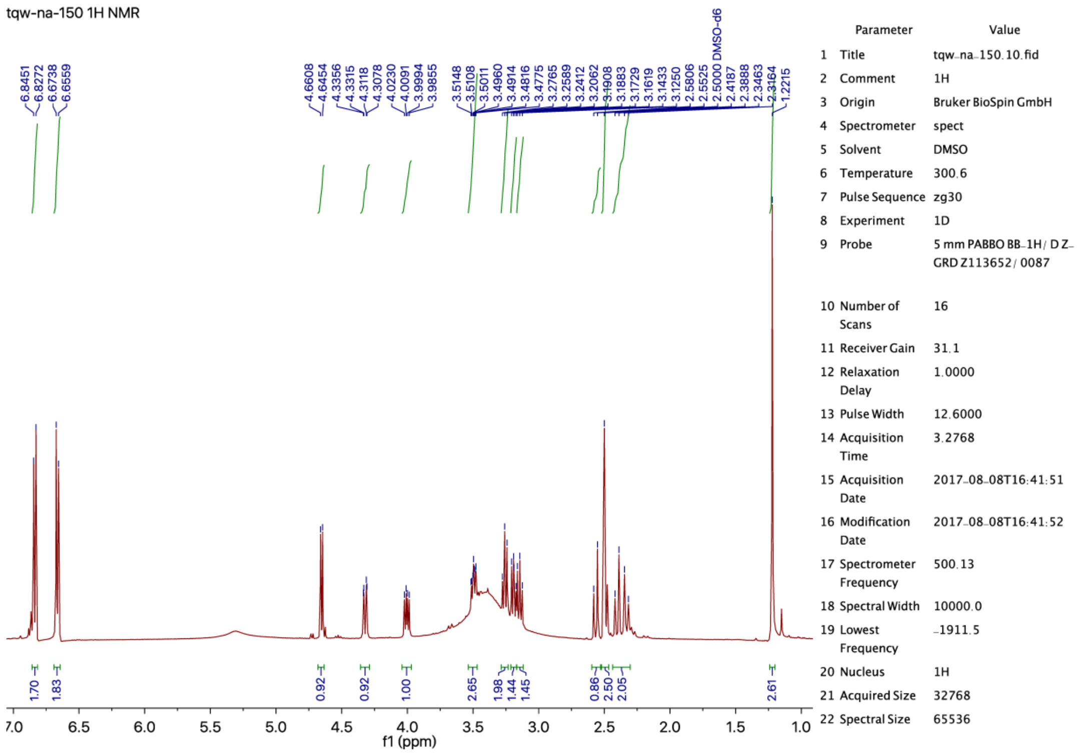

S33. ^{1}H-NMR spectra of 4-hydroxyphenyl-1-*O*-[6-(hydrogen 3-hydroxy-3-methylpentanedioate)]-β-D-glucopyranoside (5) (500 MHz, DMSO-d_6)

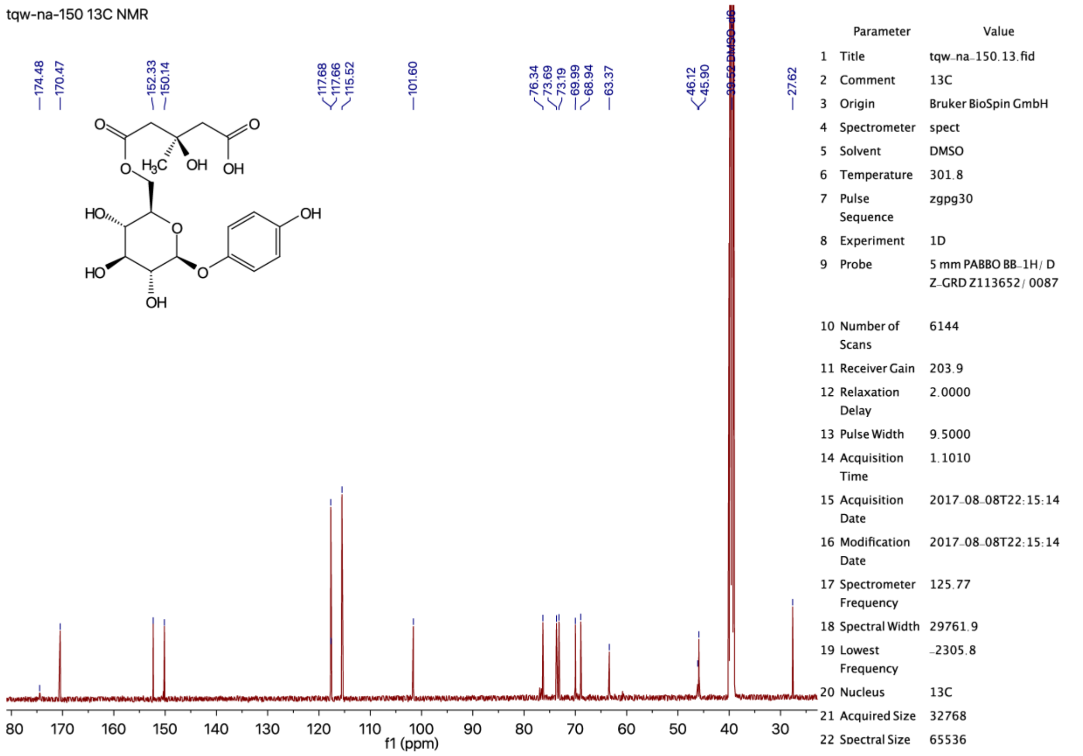

S34. ^{13}C-NMR spectra of 4-hydroxyphenyl-1-*O*-[6-(hydrogen 3-hydroxy-3-methylpentanedioate)]-β-D-glucopyranoside (5) (125 MHz, DMSO-d_6)

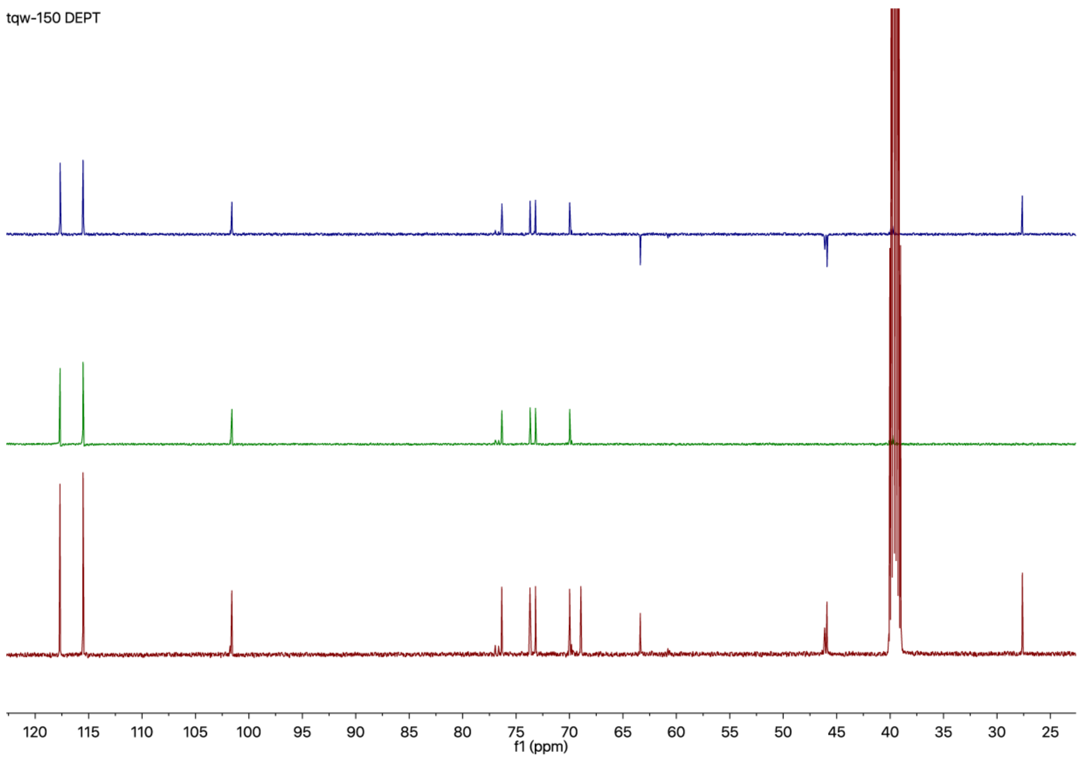

S35. DEPT of 4-hydroxyphenyl-1-*O*-[6-(hydrogen 3-hydroxy-3-methylpentanedioate)]-β-D-glucopyranoside (5)

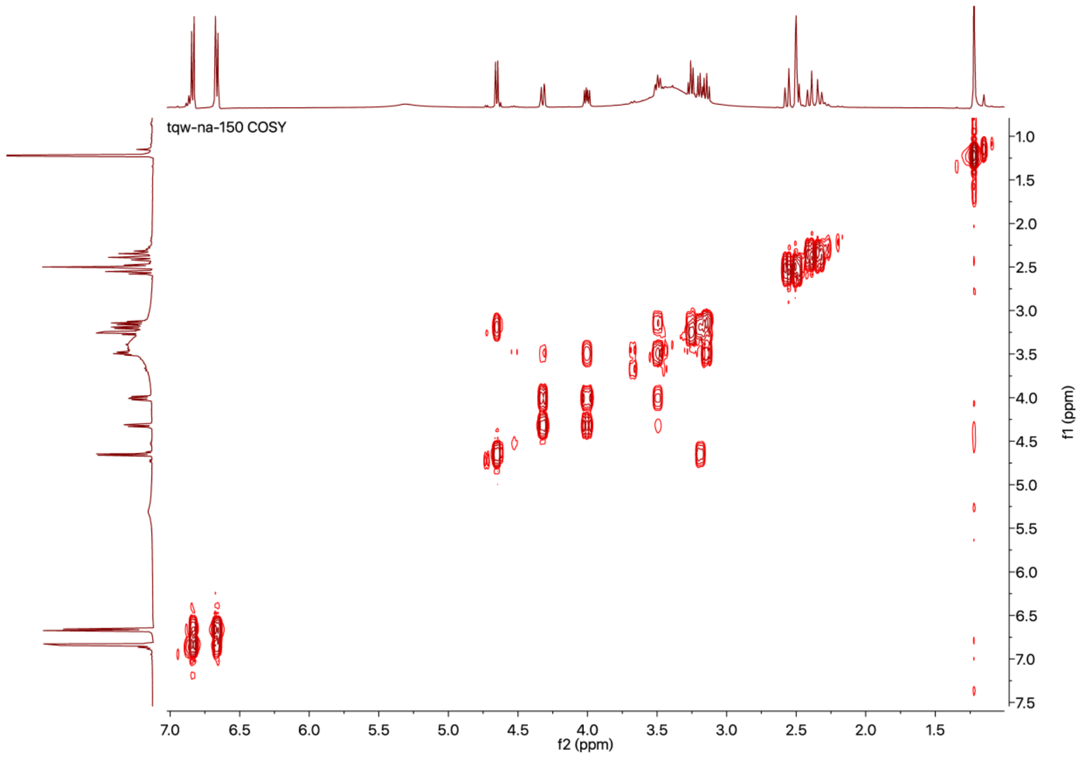

S36. ^{1}H-^{1}H COSY of 4-hydroxyphenyl-1-*O*-[6-(hydrogen 3-hydroxy-3-methylpentanedioate)]-β-D-glucopyranoside (5)

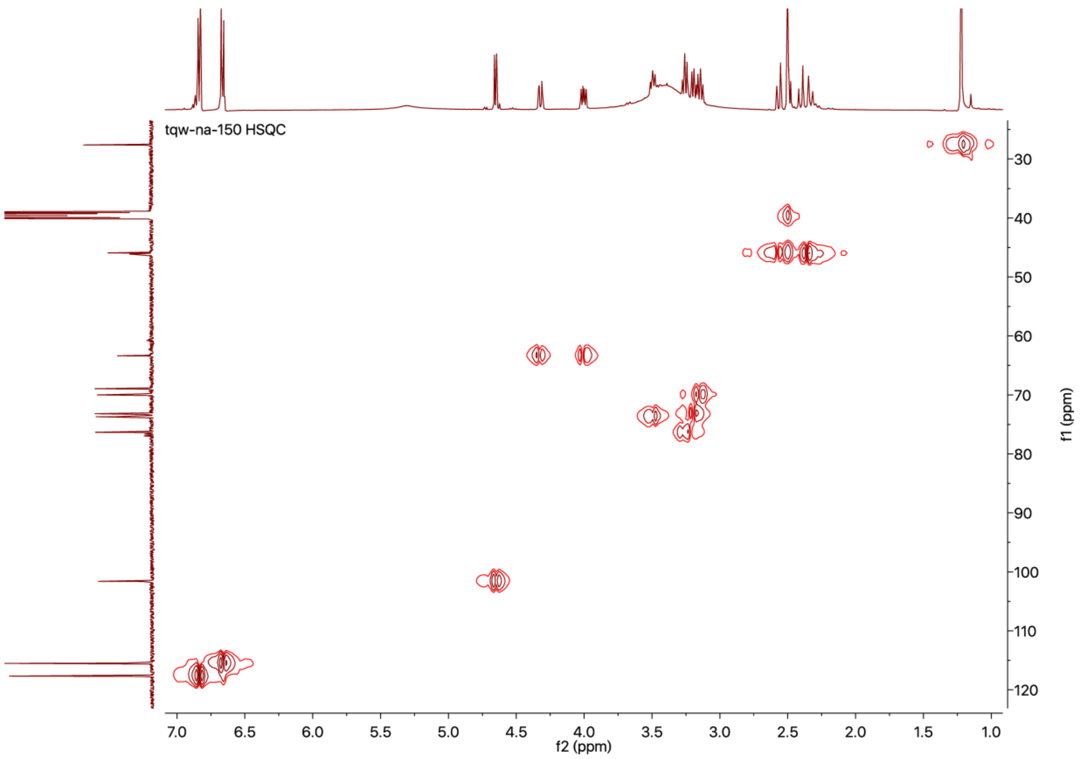

S37. HSQC of 4-hydroxyphenyl-1-*O*-[6-(hydrogen 3-hydroxy-3-methylpentanedioate)]-β-D-glucopyranoside (5)

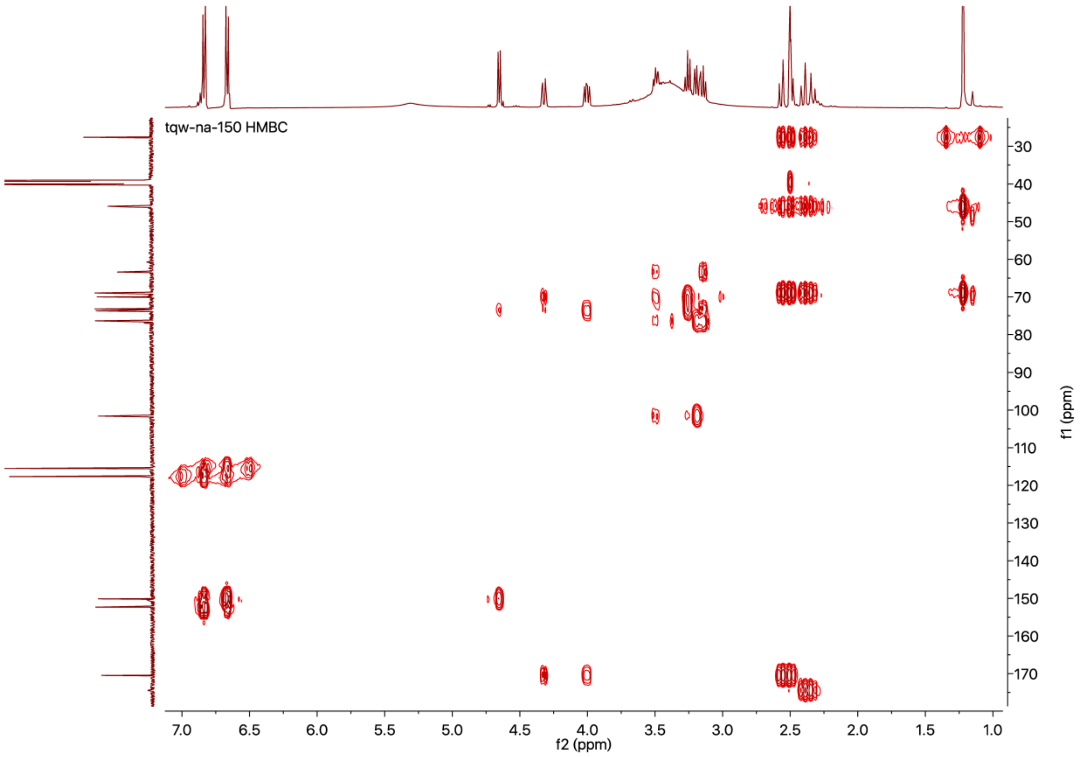

S38. HMBC of 4-hydroxyphenyl-1-*O*-[6-(hydrogen 3-hydroxy-3-methylpentanedioate)]-β-D-glucopyranoside (5)

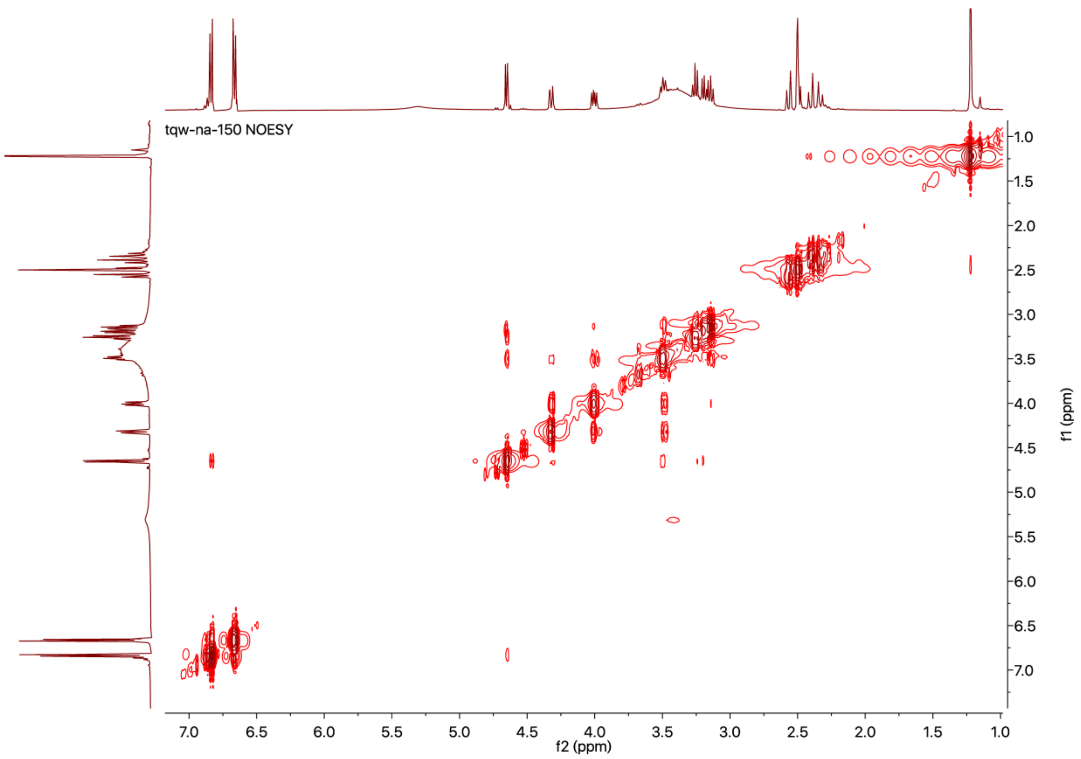

S39. NOESY of 4-hydroxyphenyl-1-*O*-[6-(hydrogen 3-hydroxy-3-methylpentanedioate)]-β-D-glucopyranoside (5)

S40. ^{1}H- and ^{13}C-NMR data of compounds 6–10 (*δ* in ppm, *J* in Hz)

Position	6[a]		7[b]		8[a]		9[a]		10[a]	
	δ_C	δ_H (*J*)	δ_C	δ_H (*J*)	δ_C	δ_H (*J*)	δ_C	δ_H (*J*)	δ_C	δ_H
1	152.4		150.5		151.3		132.8		126.0	
2, 6	119.4	7.00, d (9.0)	117.6	6.89, d (9.0)	116.8	6.60, s	130.2	7.08, d (8.6)	130.6	7.12, d (8.6)
3, 5	116.6	6.73, d (9.0)	115.6	6.65, d (9.0)	116.8	6.60, s	116.2	6.75, d (8.6)	115.7	6.76, d (8.6)
4	153.8		152.1		151.3		157.1		155.0	
7							51.2	2.87, p (6.7)	40.4	3.55, s
8							65.2	3.83, dd (10.9, 6.9)	172.9	
								3.73, dd (10.9, 6.5)		
9							65.2	3.83, dd (10.9, 6.9)		
								3.73, dd (10.9, 6.5)		
OCH_3-4									52.3	3.69, s
Glc-1′	103.6	4.77, d (7.2)	101.6	4.62, d (7.5)						
2′	75.0	3.38–3.50 (overlap)	73.3	2.91–3.25 (overlap)						
3′	78.1	3.38–3.50 (overlap)	76.9	3.40, m						
4′	71.4	3.38–3.50 (overlap)	70.2	2.91–3.25 (overlap)						
5′	78.0	3.38–3.50 (overlap)	76.5	2.91–3.25 (overlap)						
6′	62.6	3.92, dd (12.1, 1.6)	68.5	3.97, dd (11.6, 1.6)						
		3.73, dd (12.1, 5.3)		3.57, dd (11.6, 6.7)						
Glc-1″			103.3	4.21, d (7.8)						
2″			73.6	2.91–3.25 (overlap)						
3″			76.7	2.91–3.25 (overlap)						
4″			69.8	2.91–3.25 (overlap)						
5″			75.8	2.91–3.25 (overlap)						
6″			61.1	3.65, m						
				3.48, m						

[a] Recorded in CD_3OD at 500 MHz (^{1}H) and 125 MHz (^{13}C). [b] Recorded in DMSO-d_6 at 500 MHz (^{1}H) and 125 MHz (^{13}C).

S41. ^{1}H- and ^{13}C-NMR data of compounds 11–16 (δ in ppm, J in Hz)

Position	11[a]		12[a]		13[a]		14[a]		15[a]		16[a]	
	δ_C	δ_H (J)	δ_C	δ_H (J)	δ_C	δ_H (J)	δ_C	δ_H (J)	δ_C	δ_H (J)	δ_C	δ_H
1	122.7		123.1		123.1		122.0		121.4		120.7	
2	133.0	7.84, d (8.8)	117.7	7.38[a]	115.8	7.52, d (1.9)	110.3	7.05, s	110.0	7.05, s	110.5	7.14, s
3	116.0	6.78, d (8.8)	146.0	7.37, dd (8.1, 2.1)	148.6	7.52, dd (8.8, 1.9)	146.4		146.5		146.5	
4	163.3		151.5		152.6		139.6		139.7		140.3	
5	116.0	6.78, d (8.8)	115.7	6.74, d (8.1)	113.8	6.80, d (8.8)	146.4		146.5		146.5	
6	133.0	7.84, d (8.8)	123.9		125.3		110.3	7.05, s	110.0	7.05, s	110.5	7.14, s
7	170.1		170.3		170.0		170.4		169.0		167.0	
Glc-1′											95.9	5.67, d (8.0)
2′											74.1	3.37–3.52 (overlap)
3′											78.2	3.37–3.52 (overlap)
4′											71.1	3.37–3.52 (overlap)
5′											78.8	3.37–3.52 (overlap)
6′											62.3	3.86, dd (12.2, 1.9)
												3.71, dd (12.2, 4.6)
OCH_3–3					56.4	3.85, s						
OCH_3–7									52.2	3.81, s		

[a] Recorded in CD_3OD at 500 MHz (^{1}H) and 125 MHz (^{13}C).

S42. ^{1}H- and ^{13}C-NMR data of compounds 17 and 18 (δ in ppm, J in Hz)

Position	17[a]		Position	18[a]	
	δ_C	δ_H (J)		δ_C	δ_H (J)
1	119.2	8.47, d (9.0)	Glucose 1	94.9	6.37, d (2.1)
2	112.8	6.79, d (9.0)	2	69.3	4.00, m
3	141.0		3	71.4	4.82, m
4	133.3		4	62.4	4.47, m
4a	146.8		5	76.0	4.53, m
4b	112.4		6	64.9	4.96 (overlap)
					4.16, dd (11.0, 8.0)
6	163.8		Galloyl G-1	120.5	7.06, s
6a	112.2		G-2, 6	110.9	
7	108.1	7.41, s	G-3, 5	146.3	
8	146.4		G-4	140.3	
9	141.8		G-7	166.6	
10	144.0		Ring A-1	117.1	
10a	118.6		A-2	125.3	
			A-3	110.2	6.70, s
			A-4	145.5	
			A-5	138.1	
			A-6	145.2	
			A-7	168.4	
			Ring B-1	116.5	
			B-2	125.4	
			B-3	108.3	6.67, s
			B-4	145.9	
			B-5	137.5	
			B-6	145.1	
			B-7	170.0	

[a] Recorded in CD_3OD at 500 MHz (^{1}H) and 125 MHz (^{13}C).

S43. ^{1}H- and ^{13}C-NMR data of compounds 19, 20, and 27 (δ in ppm, J in Hz)

Position	**19**[a]		**20**[a]		**27**[a]	
	δ_C	δ_H (J)	δ_C	δ_H (J)	δ_C	δ_H (J)
2	158.6		158.6		158.5	
3	135.5		135.5		135.6	
4	179.5		179.4		179.4	
5	163.1		163.0		163.0	
6	100.0	6.25, d (2.1)	100.0	6.21, d (2.1)	99.9	6.22, d (2.1)
7	166.2		166.2		166.0	
8	94.8	6.45, d (2.1)	95.0	6.41, d (2.1)	94.9	6.41, d (2.1)
9	159.1		159.4		159.3	
10	105.7		105.6		105.6	
1′	122.8		122.8		123.6	
2′	132.3	8.10, d (8.9)	132.4	8.07, d (8.9)	116.1	7.68, d (2.2)
3′	116.1	6.94, d (8.9)	116.1	6.90, d (8.9)	145.8	
4′	161.6		161.5		149.8	
5′	116.1	6.94, d (8.9)	116.1	6.90, d (8.9)	117.7	6.89, d (8.4)
6′	132.3	8.10, d (8.9)	132.4	8.07, d (8.9)	123.1	7.64, dd (8.4, 2.2)
Glc-1″	104.1	5.29, d (7.4)	104.6	5.13, d (7.2)	104.7	5.12, d (7.7)
2″	75.8	3.33–3.51 (overlap)	75.8	3.24–3.55 (overlap)	75.7	3.24–3.52 (overlap)
3″	78.1	3.33–3.51 (overlap)	78.1	3.24–3.55 (overlap)	78.2	3.24–3.52 (overlap)
4″	71.4	3.33–3.51 (overlap)	71.4	3.24–3.55 (overlap)	71.4	3.24–3.52 (overlap)
5″	78.4	3.25, ddd (9.7, 5.5, 2.4)	77.2	3.24–3.55 (overlap)	77.2	3.24–3.52 (overlap)
6″	62.7	3.73, dd (11.9, 2.3)	68.6	3.81, dd (10.9, 1.3)	68.6	3.82, dd (11.1, 1.6)
		3.57, dd (11.9, 5.5)		3.24–3.55 (overlap)		3.24–3.52 (overlap)
Rha-1‴			102.4	4.52, d (1.7)	102.4	4.53, d (1.7)
2‴			72.3	3.64, dd (3.5, 1.7)	72.1	3.65, dd (3.5, 1.7)
3‴			72.1	3.53, dd (9.5, 3.5)	72.2	3.55, dd (9.5, 3.5)
4‴			73.9	3.24–3.55 (overlap)	73.9	3.24–3.52 (overlap)
5‴			69.7	3.24–3.55 (overlap)	69.7	3.24–3.52 (overlap)
6‴			17.9	1.13, d (6.2)	17.9	1.13, d (6.2)

[a] Recorded in CD_3OD at 500 MHz (^{1}H) and 125 MHz (^{13}C).

S44. ^{1}H- and ^{13}C-NMR data of compounds 21 and 28 (δ in ppm, J in Hz)

Position	21[a]		28[a]	
	δ_C	δ_H (J)	δ_C	δ_H (J)
2	158.4		158.3	
3	134.6		134.8	
4	179.0		179.0	
5	163.1		163.1	
6	99.8	6.16, d (2.1)	99.7	6.17, d (2.1)
7	165.7		165.6	
8	94.7	6.34, d (2.1)	94.7	6.34, d (2.1)
9	158.7		158.7	
10	105.8		105.8	
1′	122.9		123.5	
2′	132.2	7.96, d (8.9)	116.2	7.56, d (2.2)
3′	116.2	6.86, d (8.9)	145.8	
4′	161.3		149.6	
5′	116.2	6.86, d (8.9)	117.3	6.84, d (8.3)
6′	132.2	7.96, d (8.9)	123.2	7.54 dd (8.3, 2.2)
Glc-1″	100.6	5.64, d (8.0)	100.7	5.63, d (8.0)
2″	75.9	5.08, dd (9.6, 8.0)	75.9	5.11, dd, (9.5, 8.0)
3″	77.4	3.64, t (9.2)	77.4	3.64 (overlap)
4″	71.9	3.23–3.51 (overlap)	71.8	3.24–3.52 (overlap)
5″	76.3	3.23–3.51 (overlap)	76.4	3.24–3.52 (overlap)
6″	68.3	3.88, br d (9.7)	68.3	3.88, br d (9.9)
		3.23–3.51 (overlap)		3.24–3.52 (overlap)
Galloyl 1‴	121.6		121.5	
2‴, 6‴	110.6	7.15, s	110.6	7.16, s
3‴, 5‴	146.3		146.3	
4‴	139.8		139.8	
7‴	167.7		167.9	
Rha-1⁗	102.3	4.54, d (1.7)	102.3	4.56, d (1.6)
2⁗	72.1	3.63, dd (3.5, 1.7)	72.1	3.64 (overlap)
3⁗	72.3	3.53, dd (9.5, 3.5)	72.3	3.55, dd (9.5, 3.5)
4⁗	73.9	3.23–3.51 (overlap)	73.9	3.24–3.52 (overlap)
5⁗	69.8	3.23–3.51 (overlap)	69.8	3.24–3.52 (overlap)
6⁗	17.9	1.12, d (6.2)	17.9	1.13, d (6.2)

[a] Recorded in CD_3OD at 500 MHz (^{1}H) and 125 MHz (^{13}C)

S45. ^{1}H- and ^{13}C-NMR data of compounds 22–26 (*δ* in ppm, *J* in Hz)

Position	22[a]		23[b]		24[b]		25[b]		26[b]	
	δ_C	δ_H (*J*)	δ_C	δ_H (*J*)	δ_C	δ_H (*J*)	δ_C	δ_H (*J*)	δ_C	δ_H (*J*)
2	146.8		158.5		158.5		158.2		158.3	
3	135.7		135.6		136.2		134.9		135.3	
4	175.9		179.5		179.6		179.1		179.3	
5	160.7		163.1		163.2		163.1		162.8	
6	98.2	6.18, d (2.1)	99.9	6.22, d (2.1)	99.8	6.23, d (2.1)	99.7	6.18, d (2.1)	99.9	6.17, br s
7	163.9		166.0		165.9		165.6		165.8	
8	93.4	6.40, d (2.1)	94.7	6.41, d (2.1)	94.7	6.40, d (2.1)	94.5	6.35, d (2.1)	94.8	6.33, br s
9	156.1		159.0		159.3		158.3		159.3	
10	103.0		105.7		105.9		105.9		105.5	
1′	122.0		123.2		123.0		123.3		123.5	
2′	115.1	7.68, d (2.2)	116.0	7.73, d (2.2)	116.4	7.37, d (2.1)	116.1	7.59, d (2.2)	115.9	7.54 (overlap)
3′	145.1		145.9		146.4		145.9		145.7	
4′	147.7		149.8		149.8		149.6		149.7	
5′	115.6	6.88, d (8.5)	117.6	6.89, d (8.5)	117.0	6.94, d (8.3)	117.1	6.85, d (8.4)	117.2	6.71, d (8.4)
6′	120.0	7.54, dd (8.5, 2.2)	123.1	7.60, dd (8.5, 2.2)	122.9	7.33, dd (8.3, 2.1)	123.1	7.55, dd (8.4, 2.2)	123.0	7.54 (overlap)
1″			104.3	5.27, d (7.6)	103.5	5.38, d (1.7)	100.5	5.77, d (7.9)	104.2	5.20, d (7.7)
2″			75.7	3.50, dd (9.2, 7.6)	72.0	4.25, dd (3.4, 1.7)	76.0	5.16, dd (9.5, 7.9)	75.7	3.42–3.55 (overlap)
3″			78.4	3.37, t (9.2)	72.1	3.78, dd (9.4, 3.4)	76.4	3.69, t (9.2)	78.0	3.42–3.55 (overlap)
4″			71.2	3.45, t (8.9)	73.3	3.38 (overlap)	71.6	3.48, t (9.4)	71.4	3.42–3.55 (overlap)
5″			78.1	3.24, ddd, (9.7, 5.3, 2.4)	71.9	3.45, dq (9.6, 6.1)	78.7	3.36, ddd (9.4, 5.6, 2.3)	75.8	3.42–3.55 (overlap)
6″			62.6	3.73, dd (11.9, 2.4)	17.6	0.97, d (6.1)	62.5	3.82, dd (12.1, 2.3)	64.3	4.34, m
				3.59, dd (11.9, 5.3)				3.64, dd (12.1, 5.6)		4.27, br d (11.6)
1‴							121.5		121.2	
2‴, 6‴							110.6	7.15, s	110.2	6.94, s
3‴, 5‴							146.3		146.6	
4‴							139.8		139.7	
7‴							167.8		168.2	

[a] Recorded in DMSO-d_6 at 500 MHz (^{1}H) and 125 MHz (^{13}C). [b] Recorded in CD_3OD at 500 MHz (^{1}H) and 125 MHz (^{13}C).
